# Bipolar Hydrogen Production from a Hybrid Alkaline‐Acidic Formaldehyde‐Proton Fuel Cell

**DOI:** 10.1002/advs.202522899

**Published:** 2026-01-20

**Authors:** Feifan Liu, Lun He, Lvlv Ji, Yanjun Wen, Tao Wang, Sheng Wang

**Affiliations:** ^1^ School of Materials Science and Engineering Zhejiang Sci‐Tech University Hangzhou China; ^2^ Institute of Catalysis Research and Technology Karlsruhe Institute of Technology Eggenstein‐Leopoldshafen Germany

**Keywords:** electrocatalysis, electrochemical neutralization energy, formaldehyde oxidation reaction, fuel cell, hydrogen evolution reaction

## Abstract

Due to a positive standard reaction Gibbs free energy (Δ_r_ *G*_m_^θ^) of 237.1 kJ mol^−1^, electric energy input is indispensable for hydrogen production by conventional electrochemical water splitting. This energy requirement can be reduced by replacing the anodic oxygen evolution reaction to thermodynamic favorable small‐molecules oxidation reactions. In this work, anodic formaldehyde oxidation reaction (FOR) in alkaline media was paired with cathodic hydrogen evolution reaction (HER) in acidic media to establish a thermodynamically downhill system. The utilization of electrochemical neutralization energy in a hybrid alkaline‐acidic electrolyte configuration enables a further decrease in Δ_r_ *G*_m_^θ^. Therefore, the resulting hybrid alkaline‐acidic formaldehyde‐proton fuel cell (FPFC) exhibits a significantly reduced Δ_r_ *G*_m_^θ^ of −101.5 kJ mol^−1^. A bifunctional Ru‐doped Cu catalyst (Ru─Cu NTs@CM) was designed and synthesized to simultaneously promote the kinetics of acidic HER and alkaline FOR, demonstrating superior catalytic activity and durability to pristine Cu and Ru catalysts. This catalyst enabled concurrent bipolar H_2_ production and electricity generation from the assembled FPFC, reaching a peak power density of 18.3 mW cm^−2^ at 53.4 mA cm^−2^. A combination of (quasi) in situ characterizations and theoretical calculations unveiled the important mechanistic role of Ru‐doping in enhancing the Cu catalyst's activity and stability.

## Graphical abstract

A novel hybrid alkaline‐acidic formaldehyde‐proton fuel cell (FPFC) was developed by coupling alkaline formaldehyde oxidation reaction (FOR) with acidic hydrogen evolution reaction (HER). This design enables concurrent bipolar H_2_ production and electricity generation. The electricity produced by the FPFC can be used to drive the operation of FOR‐assisted water splitting, achieving a promising Faradaic efficiency of 400% for H_2_ production.

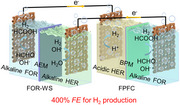

## 1 Introduction

Hydrogen (H_2_) has emerged as a promising green energy carrier owing to its high energy density, efficient energy conversion, and zero carbon emission [1, 2, 3]. Among various production methods, electrochemical water splitting powered by renewable electricity stands out as a clean and sustainable approach for H_2_ generation [4]. Conventional overall water splitting (OWS) consists of two half‐reactions: the hydrogen evolution reaction (HER) at the cathode and the oxygen evolution reaction (OER) at the anode [5]. This process is thermodynamically uphill, requiring a minimum theoretical voltage of 1.229 V corresponding to a standard reaction Gibbs free energy (Δ_r_ *G*_m_^θ^) of 237.1 kJ mol^−1^ for $H_{2}O\to H_{2}+1/2O_{2}$ [6]. To reduce energy consumption, replacing anodic OER with thermodynamic favorable small‐molecules oxidation reactions (SMORs) has emerged as a promising strategy to couple with cathodic HER [7, 8, 9, 10, 11, 12]. In alkaline media, the overall reaction of SMORs‐assisted water splitting (SMORs‐WS) can be generalized as: $H_{2}O+\left[Red\right]\to H_{2}+\left[Ox\right]$, where [*Red*] and [*Ox*] represent the reduced and oxidized species, respectively. Therefore, the Δ_r_ *G*_m_^θ^ of SMORs‐WS can be decreased when the standard Gibbs free energy of formation (Δ_f_ *G*_m_^θ^) of [*Ox*] is less than that of [*Red*] (see Supporting Information for detailed calculations). Consequently, various SMORs with lower theoretical potentials, including the oxidation of methanol, glycerol, urea, ammonia, hydrazine, furfural, etc, have been explored as energy‐efficient alternatives to OER [13].

Formaldehyde (HCHO), as a potential hydrogen carrier, has garnered increasingly research attention for H_2_ generation [14]. While thermocatalytic decomposition of HCHO over metal‐based catalysts has been widely explored for H_2_ production, recent studies have highlighted the electrocatalytic formaldehyde oxidation reaction (FOR) as a new promising alternative to OER [15, 16, 17, 18]. Notably, FOR exhibits an ultralow theoretical potential of −0.224 V vs. reversible hydrogen electrode (RHE), while simultaneously generating H_2_ and value‐added formic acid (HCOOH) at the anode ($\mathrm{HCHO}+OH^{-}\to \mathrm{HCOOH}+1/2H_{2}+e^{-}$), offering both thermodynamic and economic advantages [19]. By coupling anodic FOR with cathodic HER, bipolar H_2_ production can be achieved with a substantially reduced Δ_r_ *G*_m_^θ^ for FOR‐assisted water splitting (FOR‐WS, $\mathrm{HCHO}+H_{2}O\to \mathrm{HCOOH}+H_{2}$, Δ_r_ *G*_m_^θ^ = −21.6 kJ mol^−1^). This transformation theoretically converts the thermodynamically uphill process of conventional OWS into a downhill process in FOR‐WS. In a pioneer study, Sun and coworkers demonstrated a two‐electrode alkaline electrolyzer employing a Cu_3_Ag_7_ catalyst for FOR and a Ni_3_N/Ni catalyst for HER [19]. This system achieved bipolar H_2_ production with 200% Faradaic efficiency (*FE*), reaching an impressive current density of 100 mA cm^−2^ at an ultralow cell voltage of 0.22 V [19]. Despite these advantages, achieving industrially relevant current densities (e.g., ≥100 mA cm^−2^) inevitably requires a certain input of electricity due to unavoidable overpotentials and Ohmic losses (*IR* drop) [18, 19, 20]. Furthermore, the development of highly efficient electrocatalysts, particularly bifunctional catalysts capable of simultaneously promoting both FOR and HER kinetics, remains a significant challenge for minimizing these overpotentials and advancing practical implementation [21].

The FOR‐WS system is typically operated in alkaline media [18, 19, 20]. However, by implementing a hybrid alkaline‐acidic configuration, where FOR occurs in alkaline media and HER proceeds in acidic media, the overall reaction becomes as $\mathrm{HCHO}+OH^{-}+H^{+}\to \mathrm{HCOOH}+H_{2}$ (Δ_r_ *G*_m_^θ^ = −101.5 kJ mol^−1^). This innovative approach can further reduce the Δ_r_ *G*_m_^θ^ value by 79.9 kJ mol^−1^ compared to the typical single‐electrolyte system, with the enhancement originating from the thermodynamic driving force of the neutralization reaction ($H^{+}+OH^{-}\to H_{2}O$, Δ_r_ *G*_m_^θ^ = −79.9 kJ mol^−1^) [22]. When OH^−^ and H^+^ are selectively consumed at the anode and cathode, respectively, the neutralization reaction energy of −79.9 kJ mol^−1^ can be effectively harvested as electrochemical neutralization energy (ENE), thereby significantly reducing the overall reaction free energy [22, 23, 24]. By employing highly efficient FOR and HER catalysts, this hybrid alkaline‐acidic system can operate as a fuel cell rather than an electrolyzer, while maintaining industrially relevant current densities (≥100 mA cm^−2^) [25].

Herein, Ru‐doped Cu nanotubes in situ grown on Cu mesh (Ru─Cu NTs@CM) were fabricated by a facile synthetic method. The Ru─Cu NTs@CM catalyst shows excellent catalytic activity for both HER and FOR, which is superior to the pristine Cu and Ru catalysts. Systematic experimental and theoretical investigations elucidate that Ru‐doping modifies the electronic structure and adsorption properties of the Cu matrix, which is crucial for enhancing the catalytic performance. By integrating alkaline FOR and acidic HER, a hybrid alkaline‐acidic formaldehyde‐proton fuel cell (FPFC) of Ru─Cu NTs@CM||Ru─Cu NTs@CM was fabricated to demonstrate efficient bipolar H_2_ production and appreciable electricity output simultaneously. In addition, the electricity generated by the FPFC can power the operation of FOR‐WS, achieving a promising *FE* of 400% for H_2_ production. This work demonstrates a promising strategy for transforming a conventional endergonic water splitting electrolyzer into an exergonic bipolar H_2_‐producing fuel cell through Δ_r_ *G*_m_^θ^ modulation and tailored catalyst design.

## 2 Results and Discussion

### 2.1 Bipolar H_2_ Production Fuel Cell

**FIGURE 1:**
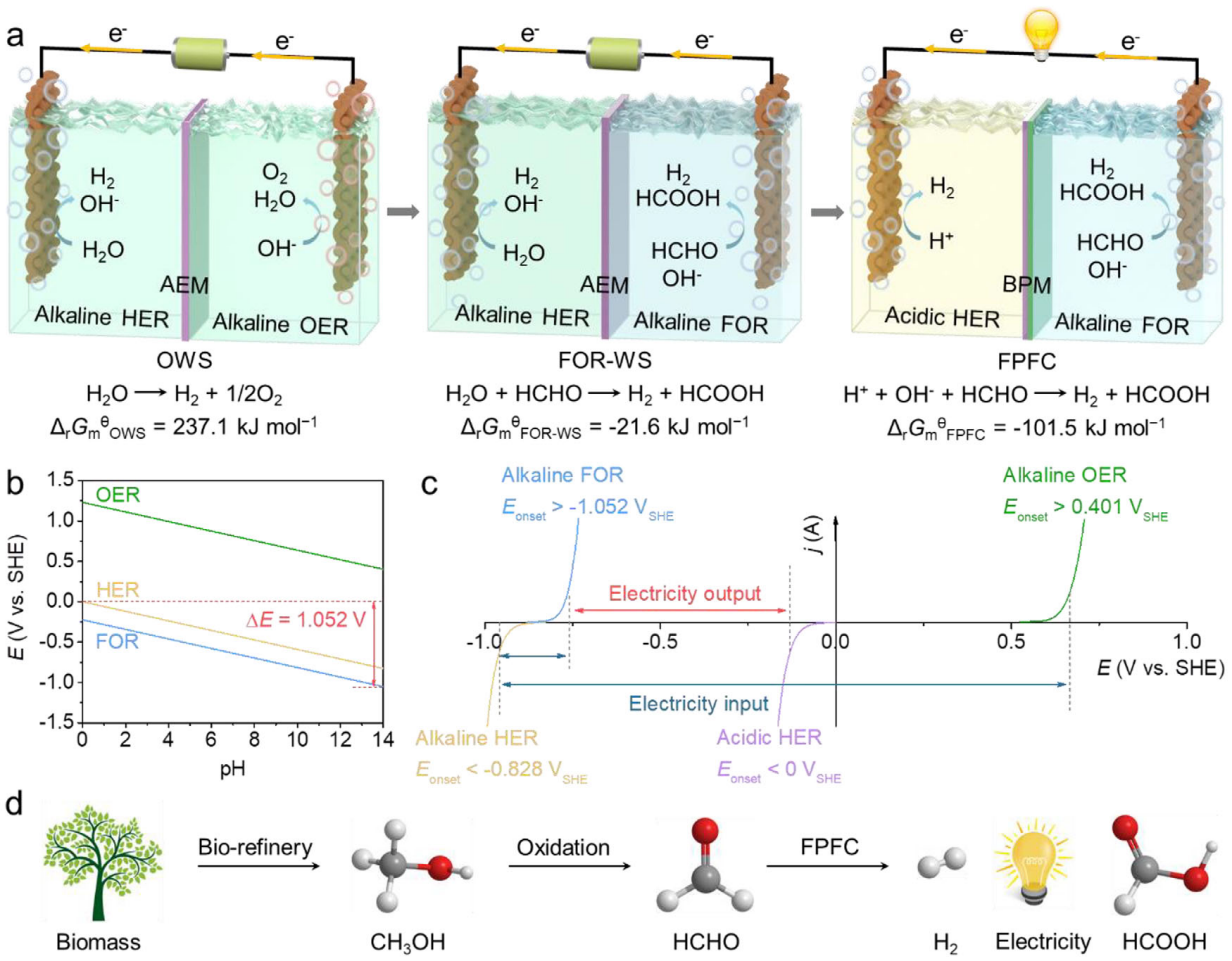
(a) Schematic illustration of OWS, FOR‐WS, and FPFC. (b) The Pourbaix diagram of HER, OER, and FOR calculated by the Nernst equation (Δ*E* = *E*_HER_(pH 0)–*E*_FOR_(pH 14) = 1.052 V). (c) Schematic LSV curves of HER, OER, and FOR in acidic and/or alkaline media. (d) The FPFC‐involved conversion route from biomass to value‐added chemicals and electricity.

Figure 1a illustrates the schematic configurations of OWS, FOR‐WS, and FPFC. Compared with conventional alkaline OWS with a positive Δ_r_ *G*_m_^θ^ of 237.1 kJ mol^−1^, FOR‐WS shows a negative Δ_r_ *G*_m_^θ^ of −21.6 kJ mol^−1^ due to the replacement of OER with FOR. Although FOR‐WS is thermodynamically spontaneous, additional electric energy input is typically indispensable to achieve an appreciable current density due to kinetic limitations. Remarkably, FPFC demonstrates an even more favorable Δ_r_ *G*_m_^θ^ of −101.5 kJ mol^−1^ by further substituting alkaline HER with acidic HER, enabled by the harvest of ENE. In FPFC, a bipolar membrane (BPM) is applied to separate the acidic and alkaline electrolytes. This Janus‐like membrane consists of an anion‐exchange membrane (AEM) and a cation‐exchange membrane (CEM) integrated back‐to‐back [26]. Figure 1b presents the Pourbaix diagram, highlighting the theoretical pH‐dependent potentials for HER, OER, and FOR. While conventional OWS requires a theoretical voltage of 1.229 V in a uniform electrolyte, FOR‐WS theoretically operates as a fuel cell but with an ultralow OCV of 0.224 V. By employing hybrid electrolytes of alkaline FOR (pH 14) at the anode and acidic HER (pH 0) at the cathode, the asymmetric FPFC achieves a significantly enhanced theoretical OCV of 1.052 V. To further clarify, Figure 1c schematically compares the linear sweep voltammetry (LSV) curves of HER, OER and FOR in acidic and/or alkaline media. Detailed thermodynamic calculations and voltage conversions are provided in the Supporting Information. The FPFC design offers significant advantages, enabling bipolar H_2_ production with a theoretical *FE* of 200%, while simultaneously generating appreciable electricity. It is noteworthy that HCHO can be industrially derived from biomass (Figure 1d), and its organic oxidation product HCOOH finds broad applications in organic synthesis, pharmaceuticals, rubber, and leather industries [27]. Thus, the FPFC‐involved conversion demonstrates a green and sustainable route for transforming biomass into value‐added chemicals and electricity.

### 2.2 Fabrication and Characterization of the Electrocatalysts

**SCHEME 1:**
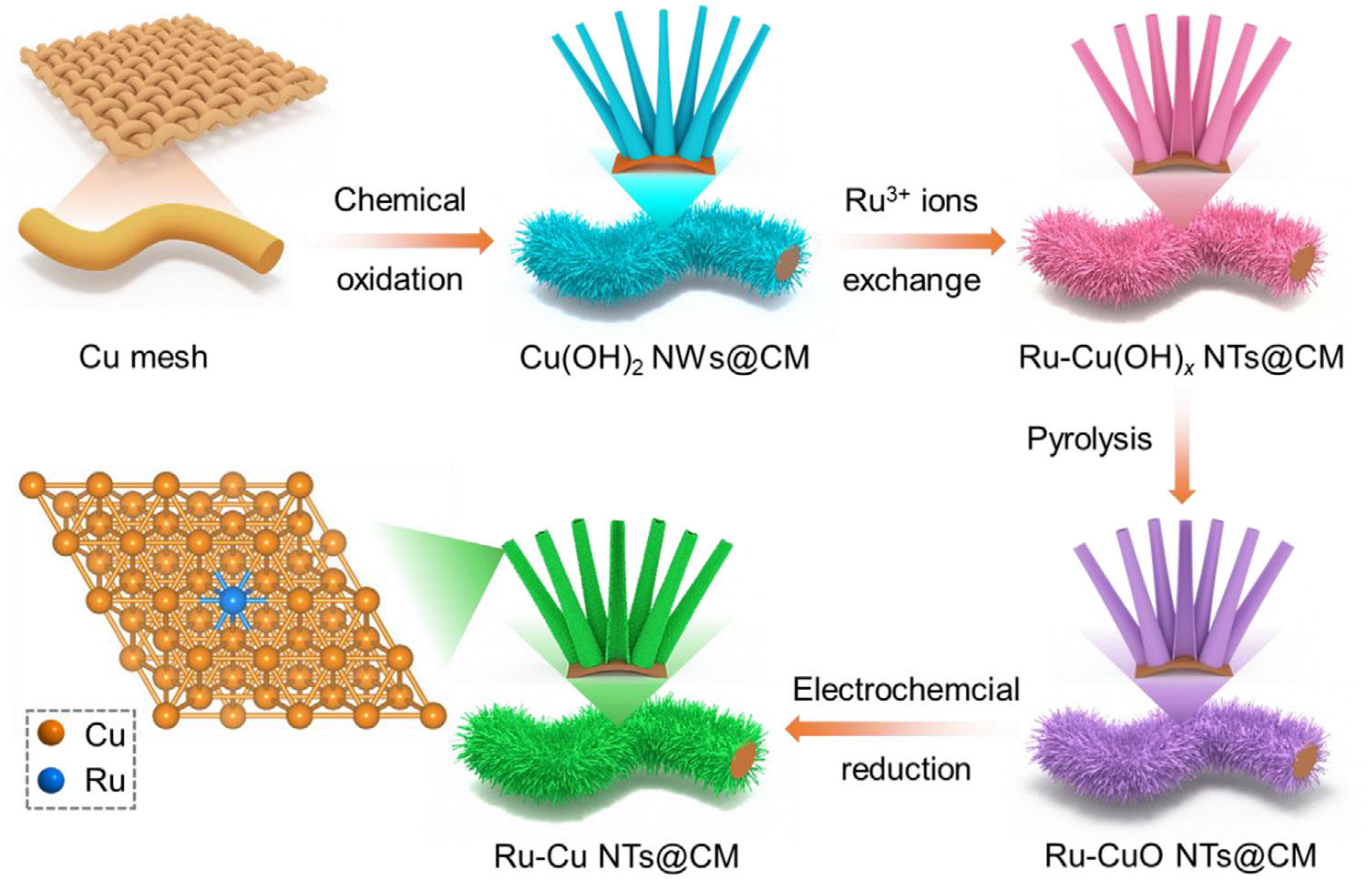
Schematic illustration of the synthetic route for Ru─Cu NTs@CM.

To achieve the enhanced electrochemical performance of FPFC, highly efficient electrocatalysts, preferably bifunctional electrocatalysts, are indispensable for both acidic HER and alkaline FOR. Scheme 1 illustrates the synthetic route for a robust electrocatalyst, Ru‐doped Cu nanotubes grown on Cu mesh (Ru─Cu NTs@CM). Typically, the surface of Cu mesh was first chemical oxidized into Cu(OH)_2_ nanowires to obtain Cu(OH)_2_ NWs@CM. The subsequent Ru^3+^ ions exchange converts solid Cu(OH)_2_ NWs into hollow Ru‐doped Cu(OH)_2_ nanotubes (Ru─Cu(OH)*_x_* NTs@CM). After undergoing the pyrolysis at 180°C in the air, Ru─Cu(OH)*_x_* NWs were oxidized into Ru‐doped CuO NTs (Ru─CuO NTs@CM). Finally, Ru─Cu NTs@CM was fabricated after the electrochemical reduction of Ru─CuO NTs@CM.

**FIGURE 2:**
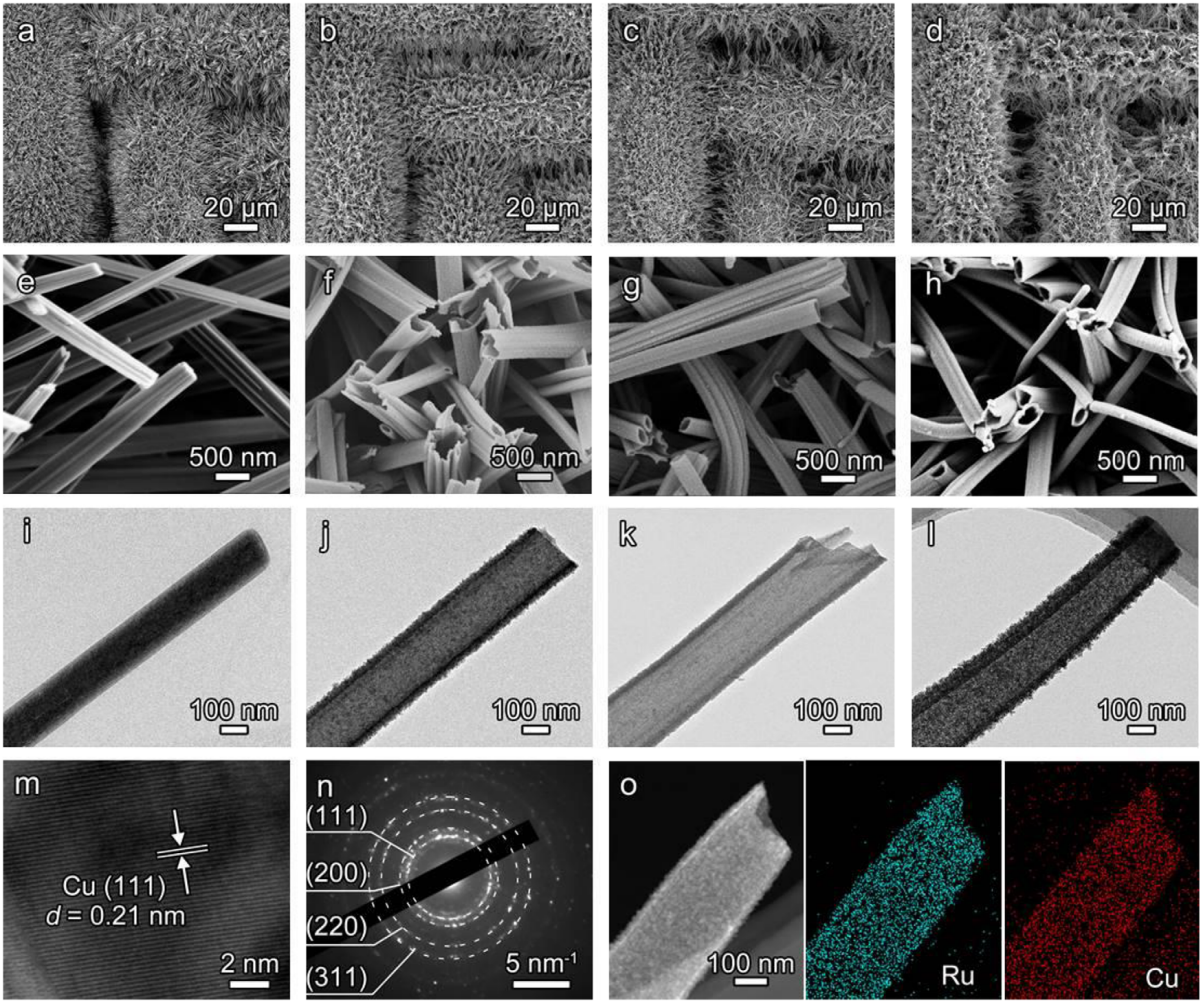
(a–h) SEM and (i–l) TEM images of (a,e,i) Cu(OH)_2_ NWs@CM, (b,f,j) Ru─Cu(OH)*_x_* NTs@CM, (c,g,k) Ru─CuO NTs@CM, and (d,h,l) Ru─Cu NTs@CM. (m) HRTEM image, (n) SAED pattern, and (o) TEM‐EDX elemental mapping images of Ru─Cu NTs@CM.

The scanning electron microscopy (SEM) images of pristine Cu mesh in Figure S1 show a smooth and clean surface. After the chemical oxidation by S_2_O_8_^2−^ ions, Cu(OH)_2_ NWs array was uniformly grown on Cu mesh (Figure 2a,e). The transmission electron microscopy (TEM) image in Figure 2i reveals that the diameter of a single Cu(OH)_2_ NW is around 160 nm. When immersing Cu(OH)_2_ NWs@CM into 5 mm RuCl_3_ solution, ions exchange between Ru^3+^ and Cu^2+^ is triggered due to the much lower *K*_sp_ of Ru(OH)_3_ (1 × 10^−36^) compared to that of Cu(OH)_2_ (4.8 × 10^−20^). The solid structured Cu(OH)_2_ NWs is gradually converted into the hollow structured Ru─Cu(OH)*_x_* NTs (Figures S2–S4). By controlling the exchange time as 30 min, a well‐defined NTs structure with an enlarged diameter of around 240 nm is obtained for Ru─Cu(OH)*_x_* NTs@CM (Figure 2b,f,j; Figure S2). After experiencing the pyrolysis, the NTs morphology is still well‐retained for Ru─CuO NTs@CM (Figure 2c,g,k). The following electroreduction of Ru─CuO NTs@CM results in the identical NTs morphology for Ru─Cu NTs@CM, except for the relative more roughened and porous surface (Figure 2d,h,l). The side‐view SEM image of Ru─Cu NTs@CM reveals that the length of Ru─Cu NTs is around 10 µm (Figure S5). The self‐supported Ru─Cu NTs array on Cu mesh is of advantageous in providing abundant active sites, benefiting efficient mass transfer and facilitating rapid electron transfer.

The SEM‐energy dispersive X‐ray analysis (SEM‐EDX) spectra in Figure S6 confirm the successful Ru^3+^ ions exchange, and the Ru feature peak is well‐maintained after the subsequent pyrolysis and electroreduction treatments. The inductively coupled plasma mass spectrometry (ICP‐MS) analysis indicates the atomic percentage of Ru content is 2.9 at% for Ru─Cu NTs@CM. In Figure 2m, the high‐resolution TEM (HRTEM) image of Ru─Cu NTs@CM shows the lattice fringe spacing of 0.21 nm, in accordance with the (111) plane of metallic Cu [16]. The selected area electron diffraction (SAED) pattern of Ru─Cu NTs@CM exhibits the multiple polycrystalline rings generating from the (111), (200), (220), and (311) crystal planes of Cu (Figure 2n). Figure 2o shows the TEM‐EDX elemental mapping images of Ru─Cu NTs@CM. Ru and Cu elements are uniformly distributed throughout a single nanotube, demonstrating the evenly doping of Ru in the Cu matrix. The compositional conversion of the overall synthetic process is further characterized by X‐ray diffraction (XRD) (Figure S7). The XRD feature peaks of the Ru species are hardly identified for Ru─Cu NTs@CM, confirming the uniform doping of Ru.

**FIGURE 3:**
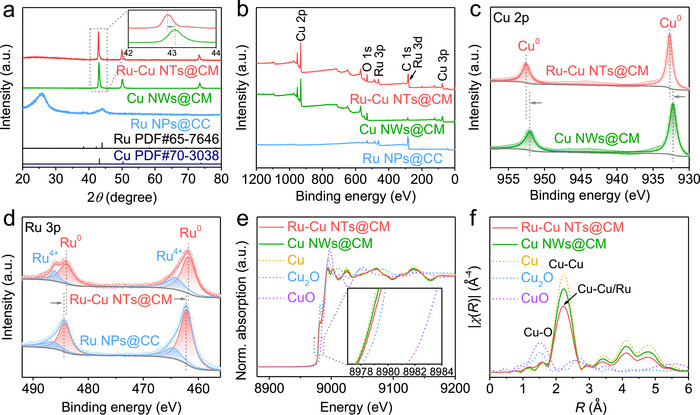
(a) XRD patterns and (b) XPS survey spectra of Ru─Cu NTs@CM, Cu NWs@CM, and Ru NPs@CC. Inset in (a) presents the enlarged XRD pattern of the rectangle region. (c) High‐resolution Cu 2p XPS spectra of Ru─Cu NTs@CM and Cu NWs@CM. (d) High‐resolution Ru 3p XPS spectra of Ru─Cu NTs@CM and Ru NPs@CC. (e) Cu *K*‐edge XANES spectra and (f) the corresponding FT‐EXAFS spectra of Ru─Cu NTs@CM, Cu NWs@CM, Cu, Cu_2_O, and CuO. Inset in (e) presents the enlarged spectra of the rectangle region.

For comparison, pristine Cu nanowires grown on Cu mesh (Cu NWs@CM) were prepared by the identical method for Ru─Cu NTs@CM, except for omitting the ions exchange step (Figure S8). In addition, metallic Ru nanoparticles grown on carbon cloth (Ru NPs@CC) were synthesized by a facile electrodeposition method (Figure S9). Figure 3a shows the XRD patterns of Ru─Cu NTs@CM, Cu NWs@CM, and Ru NPs@CC. Compared with Cu NWs@CM, the Cu diffraction peaks in Ru─Cu NTs@CM are slightly negative shifted for 0.2°, indicating the incorporation of Ru atoms into the Cu crystal lattice to form a solid‐solution alloy [18]. For Ru NPs@CC, feature peaks for metallic Ru are identified, whereas the broad peak at 25.8° is indexed into carbon cloth. In Figure 3b, the X‐ray photoelectron spectroscopy (XPS) survey spectra reveal the co‐presence of Ru and Cu in Ru─Cu NTs@CM, whereas Cu NWs@CM and Ru NPs@CC exhibit solely Cu and Ru signals, respectively. Notably, the Ru─Cu NTs@CM sample displays a +0.5 eV shift in the Cu^0^ 2p binding energy (vs. Cu NWs@CM, Figure 3c), but a −0.5 eV shift in the Ru^0^ 3p binding energy (vs. Ru NPs@CC, Figure 3d), indicating distinct electronic interactions between Cu and Ru [28]. These findings clearly demonstrate electron transfer from Cu to Ru in Ru─Cu NTs@CM, which can be attributed to the higher electronegativity of Ru (2.2 on the Pauling scale) relative to Cu (1.9) [29]. The coordination environments of Cu atoms in Ru─Cu NTs@CM and Cu NWs@CM were further probed and compared by X‐ray absorption spectroscopy (XAS). Figure 3e shows their Cu *K*‐edge X‐absorption near‐edge structure (XANES) spectra, together with the standard samples of Cu, Cu_2_O, and CuO as comparisons. The absorption edge for Ru─Cu NTs@CM is positively shifted relative to Cu NWs@CM, confirming the increase of Cu valence state after Ru doping. In Figure 3f, the corresponding Fourier transform extended X‐ray absorption fine structure (FT‐EXAFS) spectra exhibit a characteristic peak of Cu‐Cu bond at ∼ 2.2 Å for both Ru─Cu NTs@CM and Cu NWs@CM. Compared with Cu NWs@CM, the decreased Cu‐Cu peak intensity for Ru─Cu NTs@CM points to the formation of a Cu─Ru bond after the incorporation of Ru into the Cu lattice. The least‐squares EXAFS fitting curves and wavelet transform EXAFS (WT‐EXAFS) spectra of Ru─Cu NTs@CM and Cu NWs@CM are presented in Figures S10–S12 and Table S1, further revealing the Ru‐doping induced structural change at the atomic level.

### 2.3 Electrocatalytic Activity for the HER and FOR

**FIGURE 4:**
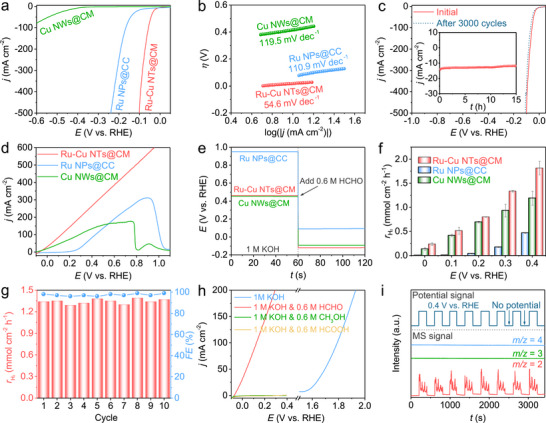
(a) LSV curves and (b) Tafel plots of electrocatalysts for HER in 0.5 m H_2_SO_4_. (c) LSV curves of Ru─Cu NTs@CM before and after 3000 CV cycles under the potential window of −0.1–0.1 V vs. RHE in 0.5 m H_2_SO_4_. The inset in (c) presents the long‐term electrolysis curve of Ru─Cu NTs@CM under the overpotential of 50 mV in 0.5 m H_2_SO_4_. (d) LSV curves of electrocatalysts for FOR in 1 m KOH and 0.6 m HCHO. (e) OCP curves of electrocatalysts in 1 m KOH with the addition of 0.6 m HCHO after 60 s. (f) H_2_ yield rates of electrocatalysts at various potentials. (g) *FEs* and H_2_ yield rates of Ru─Cu NTs@CM at 0.3 V vs. RHE during the FOR recycling measurements. (h) LSV curves of Ru─Cu NTs@CM in 1 m KOH, 1 m KOH and 0.6 m HCHO, 1 m KOH and 0.6 m CH_3_OH, and 1 m KOH and 0.6 m HCOOH. (i) DEMS signals at *m/z* = 2, 3, 4 at a pulsed potential of 0.4 V vs. RHE for Ru─Cu NTs@CM in D_2_O containing 1 m KOD and 0.6 m HCHO.

A standard three‐electrode setup was employed to evaluate the HER catalytic performances of the as‐prepared electrocatalysts in 0.5 m H_2_SO_4_. For the reference sample of Ru NPs@CC, electrodeposition time‐dependent HER catalytic performance was first investigated. Ru loading content is increasing by prolonging the electrodeposition time, but obvious crack and even exfoliation occur at a longer time (Figure S13a–h). Under 6 min of electrodeposition, Ru NPs@CC is endowed with uniform distributed Ru nanoparticles, exhibiting the optimal HER catalytic performance (Figure S13i). Therefore, it was selected as the pristine Ru reference electrocatalyst for the following comparison. Figure 4a shows the LSV curves of Ru─Cu NTs@CM, Ru NPs@CC, and Cu NWs@CM in 0.5 m H_2_SO_4_. Ru─Cu NTs@CM demonstrates the obvious superior HER catalytic activity to Ru NPs@CC and Cu NWs@CM. To achieve a current density of 10 mA cm^−2^, Ru─Cu NTs@CM requires a much lower overpotential of 17 mV compared with Ru NPs@CC (65 mV) and Cu NWs@CM (414 mV). In addition, the conductive substrates Cu mesh and carbon cloth exhibit negligible HER activity (Figure S14), indicating the surface‐grown species (Ru─Cu NTs, Ru NPs, and Cu NWs) are responsible for the catalytic activity. The Tafel plots in Figure 4b reveal the much lower Tafel slope for Ru─Cu NTs@CM (54.6 mV dec^−1^) than that for Ru NPs@CC (110.9 mV dec^−1^) and Cu NWs@CM (119.5 mV dec^−1^), suggesting the more rapid HER kinetics for the former. Moreover, the electrochemical impedance spectroscopy (EIS) measurements were conducted under the overpotential of 50 mV in 0.5 m H_2_SO_4_. The Nyquist plots in Figure S15 exhibit the lowest charge transfer resistance (*R*_ct_) for Ru─Cu NTs@CM, confirming its favorable HER catalytic kinetics. Compared with the recently reported Ru or Cu based HER electrocatalysts, the HER catalytic performance of Ru─Cu NTs@CM is also remarkable in acidic media (Table S2).

To evaluate the electrochemically active surface areas (ECSA) of the electrocatalysts, their double‐layer capacitances (*C*_dl_) were calculated by cyclic voltammetry (CV) measurements within the non‐Faradaic voltage region (Figure S16). The *C*_dl_ value is calculated as 24.3 mF cm^−2^ for Ru─Cu NTs@CM, which is higher than that for Cu NWs@CM (10 mF cm^−2^) and Ru NPs@CC (20.7 mF cm^−2^). In Figure S17, the ECSA‐normalized LSV curves point to the superiority of acidic HER intrinsic activity for Ru─Cu NTs@CM. The acidic HER catalytic durability of Ru─Cu NTs@CM was examined by both continuous CV cycling and long‐term electrolysis. As shown in Figure 4c, an obvious LSV change is hardly observed after 3000 CV cycles, and the current density is stable during 15 h of electrolysis, indicating that Ru─Cu NTs@CM possesses excellent acidic HER catalytic durability. In Figure S18, the post‐electrolysis XRD and SEM characterizations demonstrate the well‐retained crystal structure and nanotube array morphology for Ru─Cu NTs@CM, further confirming its excellent acidic HER catalytic stability. The HER catalytic performances of the electrocatalysts were further evaluated in 1 m KOH (Figures S19–S21). Ru─Cu NTs@CM shows the greatly enhanced alkaline HER catalytic activity compared with Cu NWs@CM, which is also superior to Ru NPs@CC at high overpotential (Figure S21). In addition, durability measurements and post‐electrolysis characterizations suggest good catalytic stability for Ru─Cu NTs@CM in 1 m KOH (Figure S22).

The FOR catalytic performances of the electrocatalysts were evaluated in 1 m KOH containing a certain concentration of HCHO. Figure S23 displays the LSV curves of Ru─Cu NTs@CM in 1 m KOH with different concentrations of HCHO. In 1 m KOH, a distinct anodic response is unobserved within the potential range of −0.1–0.3 V. The anodic current density rises along with the increasing concentration of HCHO, but declines when the HCHO concentration beyond 0.6 m. The optimal FOR electrolyte condition is selected as 1 m KOH and 0.6 m HCHO for further investigations. Notably, the FOR current responses under ultralow HCHO concentrations (5–25 mm) were further recorded (Figure S24a). The anodic current density increases linearly with the increase of HCHO concentration (Figure S24b), demonstrating the potential application of Ru─Cu NTs@CM as an electrochemical sensor material to detect and calculate the trace amount of HCHO in environmental solution.

Figure 4d shows the LSV curves of the electrocatalysts in 1 M KOH and 0.6 M HCHO. Ru─Cu NTs@CM and Cu NWs@CM exhibit the low anodic onset potentials of −0.07 and −0.1 V vs. RHE, respectively, whereas Ru NPs@CC shows the relative high onset potential of 0.22 V vs. RHE. It should be mentioned that the anodic FOR and cathodic HER may overlap at the potential below 0 V vs. RHE, leading to mixed current density in the LSV curve. The alkaline HER response is quite pronounced for Ru─Cu NTs@CM under the potential range of −0.1–0 V vs. RHE, whereas Cu NWs@CM is almost HER inert in this potential range (Figure S19). Therefore, the relatively lower anodic onset potential observed for Cu NWs@CM, compared to Ru─Cu NTs@CM, may be partly a result of its inferior HER activity. Above 0 V vs. RHE (and absent HER affects), Ru─Cu NTs@CM outperforms Cu NWs@CM and Ru NPs@CC, demonstrating its clear superiority. To achieve a FOR current density of 100 mA cm^−2^, Ru─Cu NTs@CM requires a much lower potential of 0.13 V vs. RHE than Cu NWs@CM (0.29 V vs. RHE) and Ru NPs@CC (0.43 V vs. RHE). Beyond 0.5 V vs. RHE, the FOR catalytic process on Cu NWs@CM is greatly affected due to the surface oxidation of Cu (Figure S25) [18]. Remarkably, Cu oxidation peaks are hardly identified in the LSV curve of Ru─Cu NTs@CM, even at a high potential of 0.95 V vs. RHE, revealing its superior FOR kinetics to rapid consume surface‐adsorbed OH^*^ and thus preventing further self‐oxidation [30]. A FOR peak is observed for Ru NPs@CC (Figure S26), indicating FOR on this catalyst is primarily controlled by mass transfer above 0.9 V vs. RHE. In contrast, the FOR current density for Ru─Cu NTs@CM climbs steadily even beyond 0.9 V vs. RHE, underscoring its rapid FOR kinetics and the mass transfer advantages conferred by its nanotube architecture. In Figure S27, the ECSA‐normalized LSV curves further reveal that Ru─Cu NTs@CM shows higher intrinsic FOR catalytic activity than Cu NWs@CM and Ru NPs@CC. In Figure S28, the EIS measurements confirm the superior FOR catalytic kinetics for Ru─Cu NTs@CM, which is evidenced by its lower *R*_ct_ than Cu NWs@CM and Ru NPs@CC. When comparing with the recently reported electrocatalysts, the FOR catalytic performance of Ru─Cu NTs@CM is also excellent (Table S3).

Open‐circuit potential (OCP) measurements were conducted to further investigate the absorption behaviours of OH^−^ and HCHO‐related species within the inner Helmholtz plane (IHP) [31]. As shown in Figure 4e, Ru─Cu NTs@CM displays a slightly higher OCP than Cu NWs@CM in 1 m KOH, indicating Ru‐doping can boost the anti‐oxidation property for Ru─Cu NTs@CM. The more noble OCP suggests that Ru‐doping alters the surface oxidation kinetics, potentially by moderating the OH^−^ adsorption process. Following the in situ introduction of 0.6 m HCHO, the OCP of Ru─Cu NTs@CM exhibits a pronounced negative shift to −0.12 V vs. RHE, a value lower than that of Cu NWs@CM (−0.09 V vs. RHE). The larger OCP drop for Ru─Cu NTs@CM (0.57 V) indicates its stronger adsorption affinity of HCHO‐related species than Cu NWs@CM (0.54 V) [31]. A key observation is that while Ru NPs@CC maintains a much higher OCP than the Cu‐based catalysts, it nonetheless undergoes the largest OCP drop (0.86 V) upon HCHO addition. This contrast demonstrates that the Ru surface possesses a stronger affinity for HCHO‐related species but a weaker affinity for OH^−^ adsorption compared to Cu, governing its interfacial chemistry. Consequently, the facilitated FOR kinetics of Ru─Cu NTs@CM are dictated by an optimal surface adsorption balance between OH^−^ and HCHO‐related species [16].

To investigate the potential‐dependent H_2_ yield rate and *FE* of FOR, electrolysis measurements were conducted at different potentials from 0 to 0.4 V vs. RHE (Figure S29). The produced H_2_ was collected and calculated by the water displacement method (Figure S30). As expected, the H_2_ yield rate is increasing with the positive shift of applied potential and Ru─Cu NTs@CM exhibits the highest rate of 1.82 mmol cm^−2^ h^−1^ at 0.4 V vs. RHE (Figure 4f). The calculated *FEs* for Ru─Cu NTs@CM are close to 100% within the potential range of 0.2–0.4 V vs. RHE (Figure S31). At the lower potential (0 or 0.1 V vs. RHE), however, the calculated *FE* is over 100% due to the influence from a spontaneous non‐Faradaic process ($HCHO+OH^{-}\to HCOO^{-}+H_{2}$) [32]. Without applying the potential, the non‐Faradaic process was further probed. The H_2_ yield rate of non‐Faradaic reaction is 0.02 mmol cm^−2^ h^−1^ for Ru─Cu NTs@CM (Figure S32), which is two orders of magnitude lower than that of electrocatalytic FOR at high potentials, making it negligible. Moreover, the (electro‐)reaction time‐dependent experiments further reveal that the contribution from non‐electrochemical H_2_ production is neglectable after 6 min of electrolysis (Figure S33). In Figure S34, the measured H_2_ yield amounts from the FOR electrolysis of Ru─Cu NTs@CM at 0.4 V vs. RHE show excellent agreement with the theoretical values, confirming that FOR is a single‐electron oxidation reaction. To quantify the organic products and evaluate the carbon balance of electrolysis, the amount of formate and methanol were determined by ^1^H nuclear magnetic resonance (NMR) spectroscopy measurement (Figure S35), and the consumption of HCHO was assessed by ultraviolet–visible (UV–vis) spectroscopy measurement (Figure S36a–c). By deducting the quantity of formate from the Cannizzaro reaction, the carbon balances are close to 100% for the FOR electrolysis of Ru─Cu NTs@CM at different potentials from 0 to 0.4 V vs. RHE (Figure S36d).

The FOR durability of Ru─Cu NTs@CM was assessed by cycling electrolysis at 0.3 V vs. RHE in 1 m KOH and 0.6 m HCHO (Figure S37). In Figure 4g, the *FEs* and H_2_ yield rates are stable for 10 cycling electrolysis, pointing to the excellent FOR durability of Ru─Cu NTs@CM. In Figure S38a,b, Ru─Cu NTs@CM exhibits negligible changes in composition and morphology after FOR electrolysis at 0.75 V vs. RHE for 10 h. In contrast, Cu NWs@CM undergoes severe self‐oxidation and structural degradation (Figure S38c,d). This remarkable contrast highlights the crucial role of Ru‐doping in enhancing the FOR durability for Cu‐based catalyst. Figure S39 shows the electrolysis curve of Ru─Cu NTs@CM in 1 m KOH with the continuous addition of 0.6 m formic acid, 0.6 m methanol, and 0.6 m HCHO. Apparaent current density can be hardly observed after the addition of formic acid or methanol, indicating their inert oxidation activities on Ru─Cu NTs@CM at 0.3 V vs. RHE. In contrast, the oxidation current density sharply rises after adding HCHO into the electrolyte, consistent with the FOR activity of Ru─Cu NTs@CM. A further comparison of the LSV curves of Ru─Cu NTs@CM in Figure 4h confirms its negligible oxidation performances for formic acid, methanol, and water at low potentials. Taken together, these results substantiate that Ru─Cu NTs@CM exhibits excellent catalytic selectivity for FOR at low potentials, which remains unaffected by the presence of formate or methanol. In Figure S40, the gas product from FOR was confirmed as H_2_ by gas chromatography. In addition, isotope‐labeled online differential electrochemical mass spectrometry (DEMS) was used to identify the origin of H atoms during the FOR. Figure 4i displays the DMES signals of FOR gaseous products in D_2_O containing 1 m KOD and 0.6 m HCHO. Only the signal of H_2_ (*m/z* = 2) was detected in response to the impulse potential, demonstrating that all H atoms are released from HCHO to produce H_2_ [16].

### 2.4 (Quasi) In situ Characterizations

**FIGURE 5:**
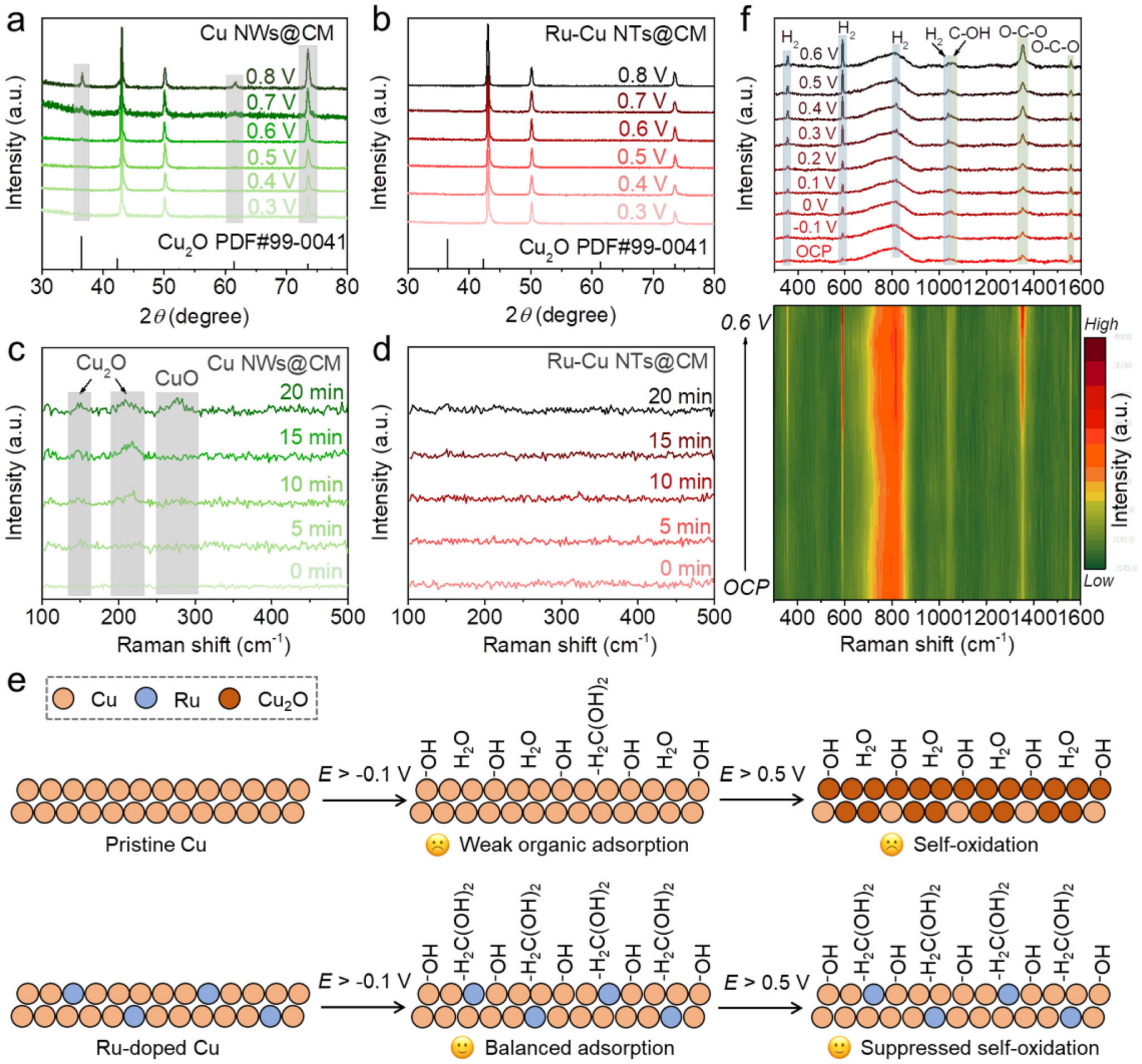
Quasi in situ XRD patterns of (a) Cu NWs@CM and (b) Ru─Cu NTs@CM after the electrolysis in 1 m KOH and 0.6 m HCHO at different potentials for 2 h. In situ Raman spectra of (c) Cu NWs@CM and (d) Ru─Cu NTs@CM recorded at 0.8 V vs. RHE in 1 m KOH and 0.6 m HCHO for different electrolysis times. (e) Schematic illustration of potential‐dependent states of Cu and Ru‐doped Cu in 1 m KOH and 0.6 m HCHO. (f) In situ Raman spectra of Ru─Cu NTs@CM recorded after the electrolysis in 1 m KOH and 0.6 m HCHO at different potentials for 5 min.

To further elucidate the crucial role of Ru‐doping in the FOR catalytic enhancement of the Cu catalyst, a series of (quasi) in situ characterizations were conducted. Figure 5a,b presents the quasi in situ XRD patterns of Cu NWs@CM and Ru─Cu NTs@CM, respectively, after 2 h of electrolysis in 1 m KOH and 0.6 m HCHO at potentials ranging from 0.3 to 0.8 V vs. RHE. For Cu NWs@CM, characteristic peaks of Cu_2_O are observed when the electrolysis potential exceeds 0.5 V vs. RHE, with their intensity enhancing at more positive potentials (Figure 5a). By contrast, feature peaks of Cu_2_O are invisible for Ru─Cu NTs@CM, even at a high electrolysis potential of 0.8 V vs. RHE (Figure 5b). At 0.8 V vs. RHE in 1 m KOH and 0.6 m HCHO, in situ Raman spectra of Cu NWs@CM reveal a continuous increase in the intensity of Cu_2_O peaks over the course of electrolysis, with CuO peak also emerging after 20 min (Figure 5c) [20]. In comparison, neither Cu_2_O and CuO feature peaks appear in the in situ Raman spectra of Ru─Cu NTs@CM (Figure 5d). All these results collectively further confirm the enhancement of the anti‐oxidation property of Ru‐doped Cu during the FOR process, especially at the high potentials.

In 1 m KOH in the absence of HCHO, however, Ru─Cu NTs@CM exhibits largely similar electrochemical property to Cu NWs@CM, albeit with a marginally enhanced resistance to oxidation (Figure S41). Typically, the electrooxidation of Cu in alkaline media involves OH^−^ adsorption on the surface, subsequent absorption into the bulk, and the dehydration of adsorbed/absorbed OH^*^ species to form Cu_2_O, followed by further oxidation to CuO (Figure S42) [33]. However, the presence of HCHO in the electrolyte can consume the surface‐adsorbed OH^*^ species, thereby initiating the FOR. For Ru─Cu NTs@CM, the optimized balance of adsorption affinities between the HCHO‐derived intermediates and OH^*^ species, along with the enhanced reaction kinetics, are critical to enabling efficient FOR while suppressing catalyst self‐oxidation (Figure 5e). The active organic species within the IHP is inferred to be hydrated HCHO (H_2_C(OH)_2_) during the FOR. The accumulation of OH^−^ ions in the electric double layer (EDL) is expected to repel negatively charged HCHO‐derived intermediates (Figure S43). Within IHP, furthermore, the proceeding of OH^−^ adsorption may facilitate the conversion of the ionized HCHO‐derived species back to H_2_C(OH)_2_.

To probe the reaction intermediates during the FOR, in situ Raman spectra of Ru─Cu NTs@CM were recorded after 5 min of FOR electrolysis, as presented in Figure 5f. The pronounced peaks at ∼ 1055 and ∼ 1352 cm^−1^ intensify with increasing electrolysis potential, which are respectively ascribed to C─OH terminal groups in H_2_C(OH)_2_^*^/H_2_COOH^*^/HCOOH^*^ and the symmetric stretching of oxygen‐bound O─C─O group in H_2_COOH^*^/HCOOH^*^ [34, 35]. The peak observed at ∼ 1555 cm^−1^ corresponds to the asymmetric O─C─O stretching of the formate product [35]. Its consistent intensity across applied potentials indicates excellent product desorption behavior on the surface of Ru─Cu NTs@CM. The four additional peaks at around 355, 587, 814, and 1035 cm^−1^ are assigned to the rotational modes of H_2_ [36]. Their intensities increase with rising potential, in accordance with the enhanced FOR rate observed at higher applied potentials.

### 2.5 DFT Calculations

**FIGURE 6:**
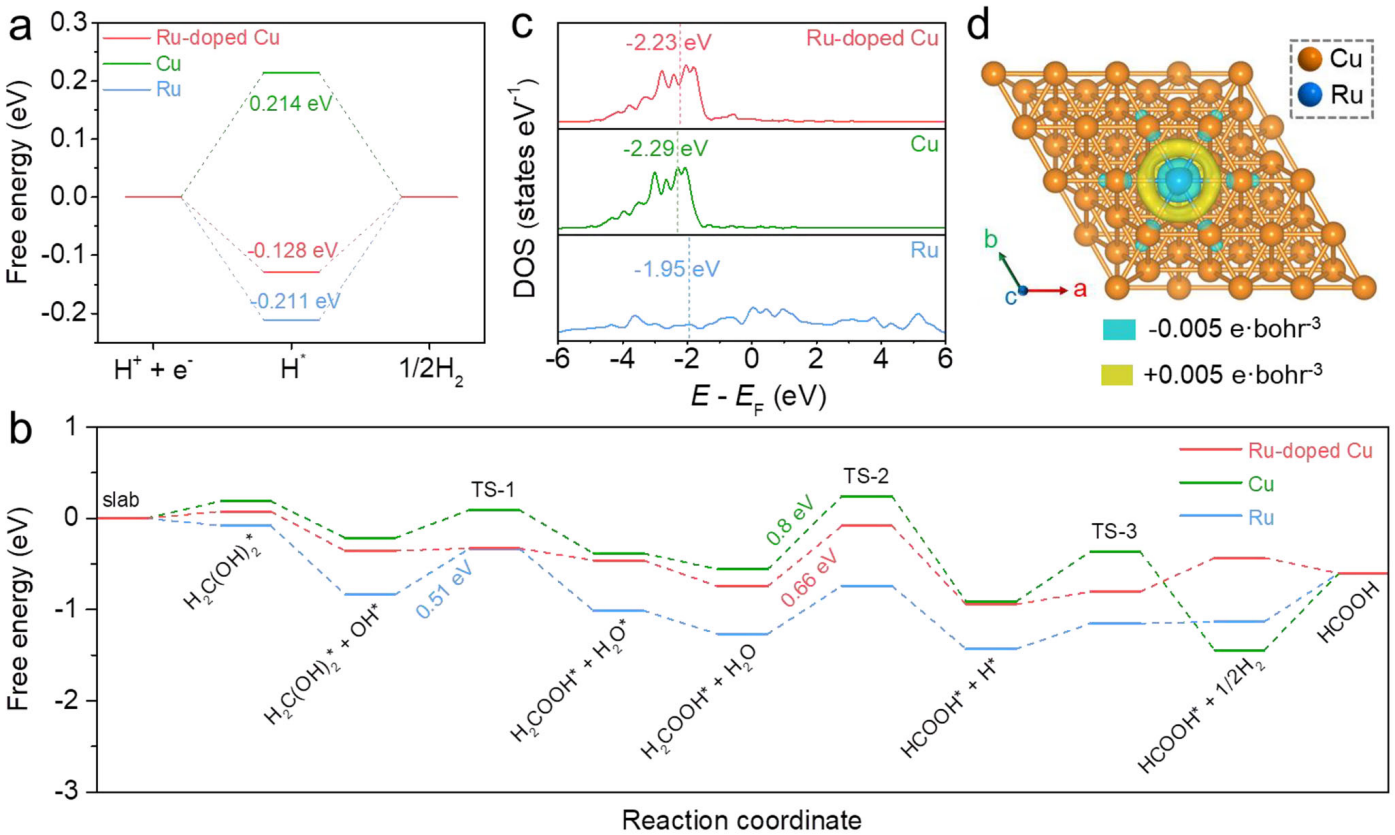
Free energy diagrams of (a) H adsorption and (b) FOR for Ru‐doped Cu, Cu, and Ru. (c) DOS of surface atoms calculated for Ru‐doped Cu, Cu, and Ru. (d) Schematic model of charge density‐difference for Ru‐doped Cu.

DFT calculations were conducted to gain insights into the mechanistic role of Ru‐doping in enhancing the HER and FOR activity for the Cu catalyst. A Cu (111) model with a single Ru atom substituted for one surface Cu atom was constructed to represent the Ru‐doped Cu system, while pristine models of Cu (111) and Ru (0001) were also constructed for comparison (Figure S44). The activity for the acidic HER was evaluated using the Gibbs free energy of hydrogen adsorption (Δ*G*_H*_), with a smaller absolute value indicating higher activity [37]. In Figure 6a, the trigonal Cu_2_Ru site on the Ru‐doped Cu surface exhibits the optimal Δ*G*_H*_ value of −0.128 eV (Figure S45), representing a significant improvement compared with pristine Cu (trigonal Cu_3_ site, 0.214 eV) and Ru (trigonal Ru_3_ site, −0.211 eV). The result is consistent with the enhanced HER activity observed for Ru─Cu NTs@CM.

Figure 6b presents the free energy diagram for the FOR pathway, accompanied by the corresponding schematic illustrations in Figures S46–S48. The proposed FOR mechanism involves the adsorption of OH^−^ with one‐electron transfer, after which the adsorbed H_2_C(OH)_2_^*^ reacts with OH^*^ to release H_2_O. The subsequent cleavage of the C─H bond produces H^*^, which then combines to form and release H_2_ [16]. For Ru‐doped Cu, Ru‐centered sites are the active sites for H_2_C(OH)_2_^*^ and the subsequent organic intermediates, while Cu is the active site for OH^*^. Doping with Ru obviously reduces the energy barrier for H_2_C(OH)_2_ adsorption to 0.08 eV from 0.18 eV on pristine Cu, although the pure Ru shows a highly favorable adsorption energy of −0.08 eV. This result directly corroborates the OCP analysis in Figure 4e. The rate‐determining step (RDS) for Ru‐doped Cu is C─H cleavage, which exhibits a lower barrier of 0.66 eV compared to 0.8 eV on pristine Cu, confirming that Ru‐doping promotes FOR intrinsic activity. The FOR RDS for pure Ru is the reaction between H_2_C(OH)_2_^*^ and OH^*^, which has a barrier of 0.51 eV. This value is even lower than the RDS barrier of Ru‐doped Cu (0.66 eV). Paradoxically, the experimental LSV curves in Figure 4d reveal a significantly higher FOR onset potential for Ru NPs@CC compared to Cu‐based catalysts. This apparent contradiction between computational and experimental results should be attributed to the initial electrochemical step of OH^−^ adsorption [38, 39]. While a previous study has shown that OH^−^ adsorption commences at a low potential of < −0.3 V vs. RHE on Cu surfaces in alkaline media [38], this process likely initiates at a more positive potential of >−0.1 V vs. RHE on Ru [39]. This shift can be rationalized by the higher potential of zero charge (PZC) of Ru compared to Cu [29]. The more positive PZC means cations (K^+^), electroneutral H_2_O and H_2_C(OH)_2_ molecules dominate the EDL (particularly IHP) on Ru at low potentials, raising the potential threshold for OH^−^ adsorption on Ru surface [40, 41]. Therefore, a higher onset potential is required for Ru NPs@CC to initiate the FOR, despite the calculated lowest RDS barrier for Ru among the models. From another perspective, this contradiction further supports the proposed catalytic mechanism.

Figure 6c displays the density of states (DOS) and the corresponding *d‐*band centers (*ε*_d_) for Ru‐doped Cu, Cu, and Ru. The *ε*_d_ value of Ru‐doped Cu is −2.23 eV, slightly higher than that of pristine Cu (−2.29 eV). This upshift in *d‐*band center enhances the bonding strength between the catalyst surface and organic intermediates, which facilitates faster FOR kinetics and helps suppress Cu self‐oxidation. Furthermore, Bader charge analysis indicates that the doped Ru atom gains 0.23 electrons from the Cu matrix (Figure 6d), consistent with the trends observed in XPS and XAS spectra. Together, all theoretical results solidly support the crucial role of Ru‐doping in modifying the electronic structure of Cu and tuning the EDL structure, ultimately enhancing the catalytic activity and stability for both HER and FOR.

### 2.6 Application for the Asymmetric FPFC

**FIGURE 7:**
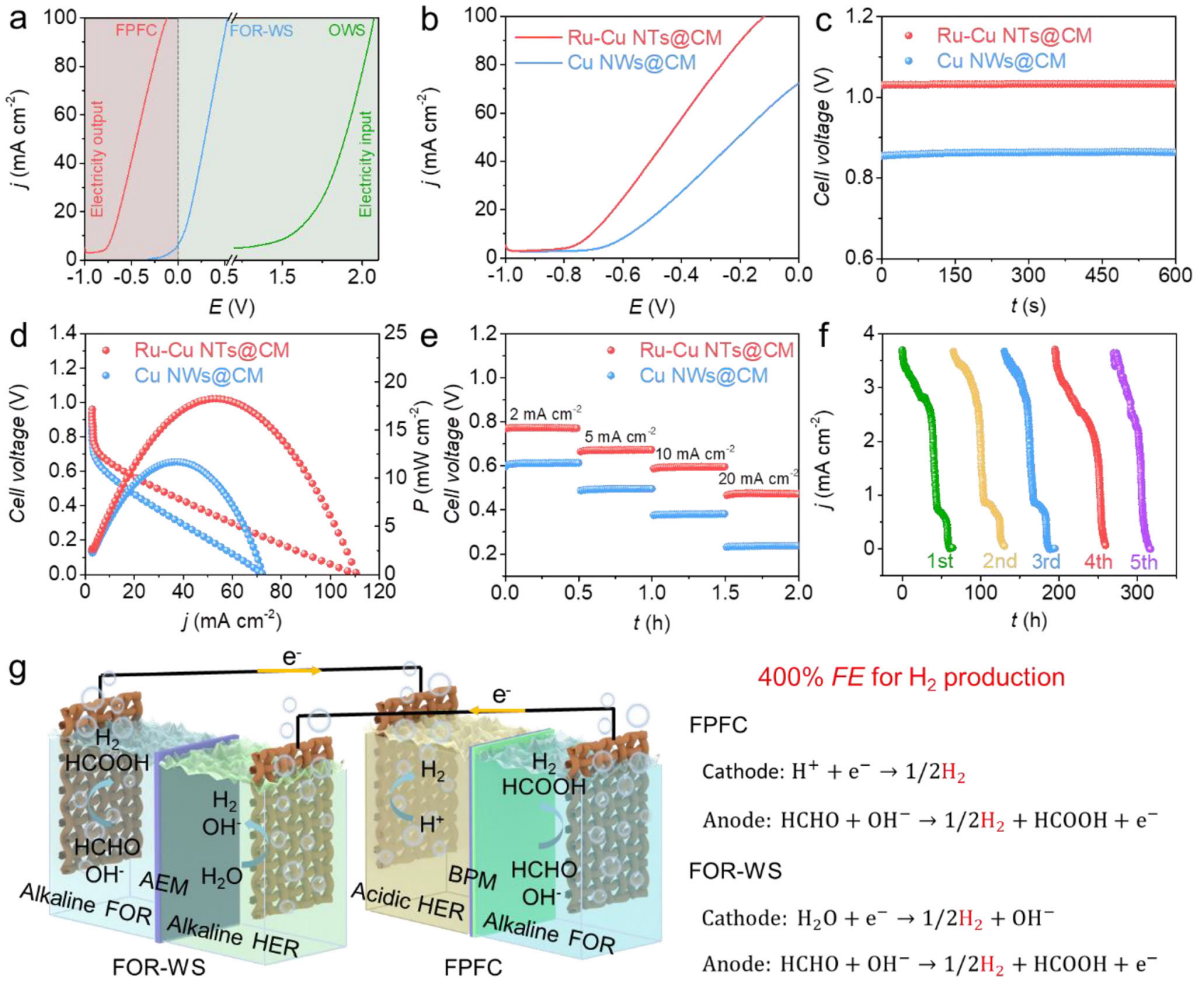
(a) LSV curves of the Ru─Cu NTs@CM||Ru─Cu NTs@CM cell/electrolyzer for FPFC, FOR‐WS, and OWS. (b) LSV curves of the Ru─Cu NTs@CM||Ru─Cu NTs@CM and Cu NWs@CM||Cu NWs@CM cells for FPFC. (c) OCVs, (d) discharge polarization curves and corresponding power density, and (e) discharge plateaus at various current densities for the Ru─Cu NTs@CM||Ru─Cu NTs@CM and Cu NWs@CM||Cu NWs@CM cells. (f) Current density measured over 5 consecutive cycles for the Ru─Cu NTs@CM||Ru─Cu NTs@CM cell using the 1000 Ω external resistors. (g) Schematic illustration of an integrated system by connecting FPFC with FOR‐WS to achieve 400% *FE* for H_2_ production.

Building on the excellent HER and FOR catalytic activity of Ru─Cu NTs@CM, a hybrid alkaline‐acidic FPFC of Ru─Cu NTs@CM||Ru─Cu NTs@CM was constructed using Ru─Cu NTs@CM as both the cathodic and anodic catalyst. The asymmetric electrolytes consist of 1 m KOH and 0.6 m HCHO and 0.5 m H_2_SO_4_ for FOR and HER, respectively, which are seperated by a BPM. For comparison, the Ru─Cu NTs@CM||Ru─Cu NTs@CM electrolyzers were also assembled for FOR‐WS in the hybrid electrolytes of 1 m KOH and 0.6 m HCHO||1 m KOH and for OWS in 1 m KOH. The LSV curves of the Ru─Cu NTs@CM||Ru─Cu NTs@CM cell/electrolyzer for FPFC, FOR‐WS, and OWS were presented in Figure 7a. To achieve the current densities of 10, 50, and 100 mA cm^−2^, the FOR‐WS mode requires significantly lower cell voltages of 0.05, 0.29, and 0.53 V, respectively, compared with 1.55, 1.89, and 2.07 V for the OWS mode. This voltage reduction is attributed to the substantially lower Δ_f_ *G*_m_^θ^ of FOR relative to OER. Notably, the FPFC mode converts the electric energy from input into output, operating at the negative voltages of −0.7, −0.45, and −0.12 V at 10, 50, and 100 mA cm^−2^, respectively. This additional voltage reduction results from the harvesting of ENE.

Compared with the Cu NWs@CM||Cu NWs@CM reference cell, the Ru─Cu NTs@CM||Ru─Cu NTs@CM cell exhibits superior performance, as revealed by the LSV curves in Figure 7b. The Ru─Cu NTs@CM||Ru─Cu NTs@CM cell exhibits a higher OCV of 1.03 V than the Cu NWs@CM||Cu NWs@CM cell (0.86 V), Figure 7c. Prior to OCV measurement, it is important to note that the dissolved O_2_ must be purged with Ar gas to prevent interference from the oxygen reduction reaction (Figure S49). In Figure 7d, the Ru─Cu NTs@CM||Ru─Cu NTs@CM cell achieves a maximum power density of 18.3 mW cm^−2^ at 53.4 mA cm^−2^, representing a significant enhancement compared to the Cu NWs@CM||Cu NWs@CM cell (11.7 mW cm^−2^ at 37.3 mA cm^−2^). The FPFC of Ru─Cu NTs@CM||Ru─Cu NTs@CM is also remarkable when comparing with the state‐of‐the‐art self‐powered H_2_ production systems in terms of *FE*, OCV, and power density (Table S4). The galvanostatic discharge profiles in Figure 7e further demonstrate the superior voltage performance of the Ru─Cu NTs@CM||Ru─Cu NTs@CM cell across various current densities. Compared to the Cu NWs@CM||Cu NWs@CM cell, the Ru─Cu NTs@CM||Ru─Cu NTs@CM cell achieves higher output voltages of 0.77, 0.67, 0.59, and 0.47 V at discharge current densities of 2, 5, 10, and 20 mA cm^−2^, respectively. By connecting three Ru─Cu NTs@CM||Ru─Cu NTs@CM cells in series, a red light‐emitting diode can be successfully illuminated, as demonstrated in Figure S50. To evaluate the operational durability, the Ru─Cu NTs@CM||Ru─Cu NTs@CM cell was operated under a constant load of 1000 Ω (Figure S51). The discharge current density was monitored throughout the test, and the electrolytes were replenished each time the current density approached zero. In Figure 7f, the discharge current density profiles exhibit high reproducibility over 5 consecutive cycles, which can be attributable to the outstanding catalytic stability of the Ru─Cu NTs@CM catalyst for both acidic HER and alkaline FOR. Interestingly, the electricity generated by the FPFC can successfully drive the operation of the FOR‐WS, thereby achieving a theoretical *FE* of 400% for H_2_ production in this integrated system (Figure 7g; Movie S1).

## 3 Conclusion

In summary, we developed a novel hybrid alkaline‐acidic FPFC system. Through thermodynamic regulation, the system achieves a favorable Δ_r_ *G*_m_^θ^ of −101.5 kJ mol^−1^, enabling simultaneous bipolar H_2_ production and electricity generation. A robust Ru─Cu NTs@CM catalyst was designed and fabricated for efficient electrocatalysis of both the HER and FOR, demonstrating superior intrinsic activity compared to pristine Cu NWs@CM and Ru NPs@CC catalysts. By synergistically leveraging both thermodynamic and kinetic modulation, the assembled Ru─Cu NTs@CM||Ru─Cu NTs@CM FPFC cell delivers remarkable performance, achieving a high OCV of 1.03 V and a maximum power density of 18.3 mW cm^−2^ at 53.4 mA cm^−2^, alongside concurrent bipolar H_2_ generation. Detailed experimental and theoretical analyses reveal that Ru‐doping is critical for enhancing the intrinsic activity and stability of the Cu‐based catalyst for HER and FOR. The enhancement mechanisms primarily involve electronic structure modification, EDL (particularly IHP) restructuring, and improved anti‐oxidation capability. Furthermore, the integration of FPFC with the FOR‐WS system achieves a favorable *FE* of 400% for H_2_ production, showcasing a pioneering strategy for highly efficient H_2_ generation.

## Conflicts of Interest

The authors declare no conflicts of interest.

## Supporting information




**Supporting File 1**: advs73866‐sup‐0001‐SuppMat.docx.

**Supporting File 2**: advs73866‐sup‐0002‐MovieS1.mp4.

## Data Availability

The data that support the findings of this study are available from the corresponding authors upon reasonable request.

## References

[1] N. Kumar , R. Aepuru , S.‐Y. Lee , and S.‐J. Park , “Recent Progress in Catalysts for Sustainable Hydrogen Production: A Comprehensive Review,” Coordination Chemistry Reviews 547 (2026): 217109.

[2] A. Odenweller and F. Ueckerdt , “The Green Hydrogen Ambition and Implementation Gap,” Nature Energy 10 (2025): 110–123.

[3] K. Bourzac , “Renewable Hydrogen is Having a Moment,” Nature Nanotechnology 20 (2025): 179–181. 10.1038/s41565-024-01838-4 39715849

[4] R. Ram , L. Xia , H. Benzidi , et al., “Water‐Hydroxide Trapping in Cobalt Tungstate for Proton Exchange Membrane Water Electrolysis,” Science 384 (2024): 1373–1380. 38900890 10.1126/science.adk9849

[5] J. Zhang , X. Fu , S. Kwon , et al., “Tantalum‐Stabilized Ruthenium Oxide Electrocatalysts for Industrial Water Electrolysis,” Science 387 (2025): 48–55. 39745949 10.1126/science.ado9938

[6] C. R. Wang , J. M. Stansberry , R. Mukundan , et al., “Proton Exchange Membrane (PEM) Water Electrolysis: Cell‐Level Considerations for Gigawatt‐Scale Deployment,” Chemical Reviews 125 (2025): 1257–1302. 39899322 10.1021/acs.chemrev.3c00904 PMC11996138

[7] L. Quan , H. Jiang , G. Mei , Y. Sun , and B. You , “Bifunctional Electrocatalysts for Overall and Hybrid Water Splitting,” Chemical Reviews 124 (2024): 3694–3812. 38517093 10.1021/acs.chemrev.3c00332

[8] B. Wu , W. Su , P. Zhu , et al., “Energy‐Saving Hydrogen Production via Small Molecules Electrooxidation‐Assisted Hybrid Systems,” Advanced Materials 37 (2025): 07842. 10.1002/adma.202507842 40686002

[9] J. Li , Y. Ma , X. Mu , et al., “Recent Advances and Perspectives on Coupled Water Electrolysis for Energy‐Saving Hydrogen Production,” Advanced Science 12 (2025): 2411964. 39777433 10.1002/advs.202411964 PMC11831450

[10] Z. Yu and L. Liu , “Recent Advances in Hybrid Seawater Electrolysis for Hydrogen Production,” Advanced Materials 36 (2024): 2308647. 10.1002/adma.202308647 38143285

[11] Z. Yu , G. D'Olimpio , H. Huang , et al., “Self‐Powered Hydrogen Production From Seawater Enabled by Trifunctional Exfoliated PtTe Nanosheet Catalysts,” Advanced Functional Materials 34 (2024): 2403099.

[12] Z. Yu , D. W. Boukhvalov , H. Tan , et al., “Sulfur and Phosphorus Co‐Doped FeCoNiCrMn High‐Entropy Alloys as Efficient Sulfion Oxidation Reaction Catalysts Enabling Self‐Powered Asymmetric Seawater Electrolysis,” Chemical Engineering Journal 494 (2024): 153094.

[13] Q. Qian , Y. Zhu , N. Ahmad , et al., “Recent Advancements in Electrochemical Hydrogen Production via Hybrid Water Splitting,” Advanced Materials 36 (2024): 2306108. 10.1002/adma.202306108 37815215

[14] J. Parthiban , M. K. Awasthi , T. A. Kharde , K. Kalita , and S. K. Singh , “Recent Progress in Molecular Transition Metal Catalysts for Hydrogen Production From Methanol and Formaldehyde,” Dalton Transactions 53 (2024): 4363–4389. 38349644 10.1039/d3dt03668e

[15] M. Ahmad , M. B. Hussain , M. A. Mushtaq , et al., “Advances in Electrocatalytic Hydrogen Evolution Coupled With Alcohol and Aldehyde Oxidation: Mechanistic Insights and Economic Feasibility,” Advanced Materials 37 (2025): 2502966. 10.1002/adma.202502966 40459514

[16] Y. Pan , Y. Li , C.‐L. Dong , et al., “Unveiling the Synergistic Effect of Multi‐Valence Cu Species to Promote Formaldehyde Oxidation for Anodic Hydrogen Production,” Chemistry 9 (2023): 963–977.

[17] Y. Zhang , J. Wu , X. Zhu , Z. Ren , and J. Chen , “L‐Arginine‐Etched Nickel‐Silver Electrocatalyst for Low‐Potential Hydrogen Evolution,” Applied Catalysis B: Environment and Energy 354 (2024): 124093.

[18] M. Yang , Y. Jiang , C. L. Dong , et al., “A Self‐Reactivated PdCu Catalyst for Aldehyde Electro‐Oxidation With Anodic Hydrogen Production,” Nature Communications 15 (2024): 9852. 10.1038/s41467-024-54286-y PMC11564531 39543188

[19] G. Li , G. Han , L. Wang , et al., “Dual Hydrogen Production From Electrocatalytic Water Reduction Coupled With Formaldehyde Oxidation via a Copper‐Silver Electrocatalyst,” Nature Communications 14 (2023): 525. 10.1038/s41467-023-36142-7 PMC9889775 36720867

[20] X. Gao , Y. Pan , J. Qiu , J. Peng , S. Wang , and Y. Zou , “Enhancing the Stability of Cu‐Based Electrocatalyst via Fe Alloy in Electrocatalytic Formaldehyde Oxidation With Long Durability,” Advanced Functional Materials 35 (2024): 2417545.

[21] J. Li , T. Zhang , Y. Ma , Z. Zhao , H. Ma , and Z. Guo , “Bipolar Hydrogen Production by Hybrid Water Electrolysis,” Advanced Functional Materials (2025): 15761.

[22] Y. Ding , P. Cai , and Z. Wen , “Electrochemical Neutralization Energy: From Concept to Devices,” Chemical Society Reviews 50 (2021): 1495–1511. 33346772 10.1039/d0cs01239d

[23] D. Yan , C. Mebrahtu , S. Wang , and R. Palkovits , “Innovative Electrochemical Strategies for Hydrogen Production: From Electricity Input to Electricity Output,” Angewandte Chemie International Edition 62 (2023): 202214333. 10.1002/anie.202214333 36437229

[24] Y. Yang , J. Xu , Y. Lai , et al., “Interfacial Engineering of RuTe_2_‐Ru for Co‐Generation of Hydrogen and Electricity,” Applied Catalysis B: Environment and Energy 358 (2024): 124414.

[25] F. Hu , K. Chen , Z. Lu , et al., “Self‐Powered Electrocatalytic Aldehyde Reforming Fuel Cell for Sustainable H_2_ Generation With ∼200% Faradaic Efficiency,” Angewandte Chemie International Edition 64 (2025): 202504894. 10.1002/anie.202504894 40231609

[26] S. Sasmal , L. Chen , P. V. Sarma , et al., “Materials Descriptors for Advanced Water Dissociation Catalysts in Bipolar Membranes,” Nature Materials 23 (2024): 1421–1427. 38951650 10.1038/s41563-024-01943-8

[27] D. S. Tran , N.‐N. Vu , H.‐E. Nemamcha , et al., “Design of Electrocatalysts and Electrodes for CO_2_ Electroreduction to Formic Acid and Formate,” Coordination Chemistry Review 524 (2025): 216322.

[28] H. Huang , H. Jung , S. Li , S. Kim , J. W. Han , and J. Lee , “Activation of Inert Copper for Significantly Enhanced Hydrogen Evolution Behaviors by Trace Ruthenium Doping,” Nano Energy 92 (2022): 106763.

[29] S. Trasatti , “Work Function, Electronegativity, and Electrochemical Behaviour of Metals,” Journal of Electroanalytical Chemistry and Interfacial Electrochemistry 33 (1971): 351–378.

[30] N. C. Ramos and A. Holewinski , “Recent Advances in Anodic Hydrogen Production: Electrochemical Oxidative Dehydrogenation of Aldehydes to Carboxylates,” Current Opinion in Electrochemistry 45 (2024): 101484.

[31] H. Yu , S. Jiang , W. Zhan , et al., “Formaldehyde Oxidation Boosts Ultra‐Low Cell Voltage Industrial Current Density Water Electrolysis for Dual Hydrogen Production,” Chemical Engineering Journal 475 (2023): 146210.

[32] E. C. Ashby , F. Doctorovich , C. L. Liotta , et al., “Concerning the Formation of Hydrogen in Nuclear Waste. Quantitative Generation of Hydrogen via a Cannizzaro Intermediate,” Journal of the American Chemical Society 115 (1993): 1171–1173.

[33] M. R. G. De Chialvo , S. L. Marchiano , and A. J. Arvía , “The Mechanism of Oxidation of Copper in Alkaline Solutions,” Journal of Applied Electrochemistry 14 (1984): 165–175.

[34] L. Du , K. Qian , X. Zhu , et al., “Interface Engineering of Palladium and Zinc Oxide Nanorods With Strong Metal–Support Interactions for Enhanced Hydrogen Production From Base‐Free Formaldehyde Solution,” Journal of Materials Chemistry A 7 (2019): 8855–8864.

[35] Z. Li , P. Wang , G. Han , et al., “Ampere‐Level Co‐Electrosynthesis of Formate From CO_2_ Reduction Paired With Formaldehyde Dehydrogenation Reactions,” Nature Communications 16 (2025): 4850. 10.1038/s41467-025-60008-9 PMC12103498 40413166

[36] I. Bonnin , R. Mereau , K. De Oliveira Vigier , and T. Tassaing , “Collision‐Induced Infrared Absorption and Raman Scattering of H_2_ in Supercritical CO_2_ ,” Journal of Molecular Liquids 360 (2022): 119455.

[37] J. K. Nørskov , T. Bligaard , A. Logadottir , et al., “Trends in the Exchange Current for Hydrogen Evolution,” Journal of the Electrochemical Society 152 (2005): 23–26.

[38] A. Tiwari , H. H. Heenen , A. S. Bjørnlund , et al., “Fingerprint Voltammograms of Copper Single Crystals Under Alkaline Conditions: A Fundamental Mechanistic Analysis,” The Journal of Physical Chemistry Letters 11 (2020): 1450–1455. 32022563 10.1021/acs.jpclett.9b03728

[39] Y. Fang , C. Wei , T. Liu , et al., “Anomalous pH‐Dependence of Ru for Hydrogen Electrochemistry,” Angewandte Chemie International Edition 64 (2025): 202508239. 10.1002/anie.202508239 40411158

[40] A. Auer , X. Ding , A. S. Bandarenka , and J. Kunze‐Liebhäuser , “The Potential of Zero Charge and the Electrochemical Interface Structure of Cu(111) in Alkaline Solutions,” The Journal of Physical Chemistry C 125 (2021): 5020–5028. 10.1021/acs.jpcc.0c09289 PMC8016203 33828636

[41] H. Wang and H. D. Abruña , “Identifying Adsorbed OH Species on Pt and Ru Electrodes With Surface‐Enhanced Infrared Absorption Spectroscopy Through CO Displacement,” Journal of the American Chemical Society 145 (2023): 18439–18446. 37552880 10.1021/jacs.3c04785

