# Supplementary material for: Bipolar Hydrogen Production from a Hybrid Alkaline‐Acidic Formaldehyde‐Proton Fuel Cell
Source: Adv Sci (Weinh). 2026 Jan 20;13(17):e22899. doi: 10.1002/advs.202522899 (PMC13042820; doi:10.1002/advs.202522899)
Supplement: Supplementary file 1 — Supporting File 1: advs73866‐sup‐0001‐SuppMat.docx. [file ADVS-13-e22899-s001.docx]

**Supporting Information**

**Bipolar Hydrogen Production from a Hybrid Alkaline-Acidic Formaldehyde-Proton Fuel Cell**

*Feifan Liu,^a^ Lun He,^a^ Lvlv Ji,^a*^ Yanjun Wen,^b*^ Tao Wang,^a^ and Sheng Wang^a*^*

^[a]^School of Materials Science and Engineering, Zhejiang Sci-Tech University, Hangzhou 310018, China

^[b]^Institute of Catalysis Research and Technology, Karlsruhe Institute of Technology, Eggenstein-Leopoldshafen 76344, Germany

^[*]^Corresponding authors. E-mail addresses: llji@zstu.edu.cn (Lvlv Ji); wen.yanjun@kit.edu (Yanjun Wen); wangsheng@zstu.edu.cn (Sheng Wang)

**Experimental section**

**Chemicals**

Ruthenium chloride trihydrate (RuCl_3_⋅3H_2_O, 98%), sodium hydroxide (NaOH, 96%), ammonium persulfate ((NH_4_)_2_S_2_O_8_, 98%), and formaldehyde solution (HCHO, 37 wt% in H_2_O, containing 10 - 15% CH_3_OH stabilizer) were purchased from Macklin Reagents Ltd. Potassium hydroxide (KOH, 99.99%) was purchased from GaoJing Chemical Plant. Sulfuric acid (H_2_SO_4_, 98%) and hydrochloric acid (HCl, 36%) were obtained from Hangzhou Shuanglin Chemical Reagent Co., Ltd. All chemicals were used as received without any further purification. Cu mesh (400 mesh, 0.56 mm thickness) was purchased from the local electronic material market. Commercial conductive carbon cloth (CC, W0S1011, thickness: 0.3 mm) was purchased from Jingke Material Technology, Beijing. High-purity Ar (99.99%) gas were purchased from Hangzhou Gases Co. All aqueous electrolyte solutions were prepared using Milli-Q ultrapure water (>18 MΩ·cm).

***Formaldehyde handling and safety protocols.*** Formaldehyde solutions were stored in a dedicated, ventilated chemical storage cabinet at room temperature and away from oxidizers or ignition sources. All experiments involving formaldehyde were conducted in a certified fume hood with continuous airflow. Researchers need to complete specific chemical safety training for handling volatile toxic compounds prior to the study. Protective mask was required to wear during the experiment. All formaldehyde-containing waste was collected in labeled, chemically compatible containers and neutralized before disposal.

**Materials synthesis**

***Synthesis of Cu(OH)_2_ NWs@CM.*** Cu mesh (0.5 cm × 1 cm) was sequentially ultrasonicated with 1 M HCl, ethanol, and deionized water for each 5 minutes to dissolve surface oxides and organic residues. The cleaned Cu mesh was then vertically immersed in 50 mL of a mixed solution containing 2.5 M NaOH and 0.15 M (NH_4_)_2_S_2_O_8_ for 30 min, followed by washing with deionized water and ethanol. The resultant Cu(OH)_2_ nanowires vertically *in situ* grown on Cu mesh (denoted as Cu(OH)_2_ NWs@CM) were dried in an oven at 60 °C for 10 min.

***Synthesis of Cu NWs@CM.*** Cu(OH)_2_ NWs@CM were first annealed in an oven at 180 °C for 40 min to get CuO NWs@CM. After that, the electroreduction process was conducted to reduce CuO to metallic Cu in 1 M KOH. In a two-electrode system, CuO NWs@CM and Pt sheet were used as working and counter electrode, respectively. Electroreduction was carried out at -3 V for 10 min. The resultant Cu nanowires grown on Cu mesh (denoted as Cu NWs@CM) were obtained after washing with deionized water and ethanol, and subsequent drying.

***Synthesis of Ru-Cu NTs@CM.*** Cu(OH)_2_ NWs@CM were first immersed in 40 mL of 5 mM RuCl_3_ aqueous solution for 30 min. Ions exchange between Ru^3+^ and Cu^2+^ was gradually occurred to form hollow nanotube structured Ru-doped Cu(OH)*_x_* (denoted as Ru-Cu(OH)*_x_* NTs@CM). In the parallel experiments, the ions exchange times were also set as 10, 20, 40 and 60 min, respectively, to investigate the time-dependent morphological and compositional conversion. After that, Ru-Cu(OH)*_x_* NTs@CM were annealed and then electrochemical reduced under the identical conditions applied for Cu(OH)_2_ NWs@CM above. The resultant Ru-doped Cu nanotubes grown on Cu mesh (denoted as Ru-Cu NTs@CM) were obtained after washing with deionized water and ethanol, and subsequent drying.

***Synthesis of Ru NPs@CC.*** Carbon cloth (0.5 cm × 1 cm) was sequentially ultrasonicated in 1 M HCl, ethanol, and deionized water for each 5 min. A two-electrode setup was constructed by employing carbon cloth as working electrode, Ru sheet as counter electrode, and the mixed solution of 10 mM RuCl_3_ and 1 M HCl as electrolyte. Electrodeposition of Ru on carbon cloth was carried out at a constant current density of -10 mA cm^−2^ for 6 min. After that, the product metallic Ru nanoparticles deposited on carbon cloth (denoted as Ru NPs@CC) was obtained after washing with deionized water and ethanol, and subsequent drying. In the parallel experiments, Ru NPs@CC-2 min, Ru NPs@CC-4 min, Ru NPs@CC-8 min and Ru NPs@CC-10 min were obtained dependent on the electrodeposition times were set as 2, 4, 8 and 10 min, respectively.

**Characterizations**

Scanning electron microscopy (SEM) images and energy dispersive X-ray analysis (EDX) data were documented on Zeiss VLTRA-55 equipped with a Horiba EDX system. Transmission electron microscopy (TEM) images, high-resolution TEM (HRTEM) images, selected area electron diffraction (SAED) patterns and EDX elemental mapping images were recorded on JEM-2010 HR. Powder X-ray diffraction (XRD) patterns were measured on Bruker D8 Focus via ceramic monochromatized Cu Kα radiation of 1.54178Å. X-ray photoelectron spectroscopy (XPS) was performed using a spectrometer (Kratos Axis Ultra DLD) operated at 12 kV. Fourier transform infrared (FT-IR) spectra were recorded by using a Nicolet 6700 spectrometer. Raman spectra were obtained on a confocal microscope laser Raman spectrometer (Reinshaw InVia) with 532 nm laser excitation. The electrolyte products were analyzed using nuclear magnetic resonance (NMR) spectroscopy on an Avance III 400 MHz NMR spectrometer (Bruker, Germany). The ultraviolet-visible (UV-Vis) spectra were collected using a Shimadzu UV - 1800 spectrophotometer. Inductively Coupled Plasma Mass Spectrometry (ICP-MS) was performed on the Agilent 7700. X-ray absorption spectroscopy (XAS) measurements were conducted at the BL14W1 beamline of the Shanghai Synchrotron Radiation Facility (SSRF), China. Data processing and analysis were conducted by Athena and Artemis software. The produced H_2_ was detected using a gas chromatography (GC7900) with LabSolar Ⅲ AG serving as the vacuum-sealed gas circulation system and Ar as the carrier gas.

**Electrochemical measurements**

Electrochemical measurements were conducted on a CHI 660E electrochemical workstation (Chenhua Corp, Shanghai, China) by using a three-electrode setup. The setup was composed of a self-supported working electrode, a carbon rod counter electrode, and a saturated calomel reference electrode (SCE). The effective contact area of working electrode in the electrolyte was controlled as 0.25 cm^2^. The hydrogen evolution reaction (HER) performances were evaluated in 0.5 M H_2_SO_4_ and 1 M KOH, the oxygen evolution reaction (OER) performances were examined in 1 M KOH, and the formaldehyde oxidation reaction (FOR) performances were assessed in 1 M KOH containing a certain concentration of HCHO. All potentials were converted to the reversible hydrogen electrode (RHE) scale using the Nernst equation (*E*_RHE_ = *E*_SCE_ + 0.0592 × *pH* + 0.244), which were experimentally calibrated considering RHE in the high-purity H_2_-saturated electrolyte with polished Pt sheets as both the working and counter electrodes. In 0.5 M H_2_SO_4_, *E*_RHE_ = *E*_SCE_ + 0.27 V; in 1 M KOH, *E*_RHE_ = *E*_SCE_ + 1.05 V. Linear sweep voltammetry (LSV) was performed at a scan rate of 2 mV s^−1^. Tafel slopes were obtained by fitting the data from the linear regions of the corresponding LSV curves to the Tafel equation. Electrochemical impedance spectroscopy (EIS) was measured over a frequency range of 10^−2^ to 10^5^ Hz with an applied potential amplitude of 5 mV. Cyclic voltammetry (CV) was employed to measure the double-layer capacitance (*C*_dl_) at different scan rates ranging from 50 to 250 mV s^−1^. Prior to the measurements, the electrolyte was purged with Ar for 30 min to remove dissolved O_2_. Unless stated otherwise, LSV and Tafel data plots were corrected with 90% *iR* compensation. For FOR, no *iR* compensation was applied. All the electrochemical measurements were conducted at 25 ± 2 ^o^C.

Overall water splitting (OWS), formaldehyde-assisted water splitting (FOR-WS), and formaldehyde-proton fuel cell (FPFC) measurements were conducted in a two-electrode setup. The as-prepared self-supported electrodes were applied as both the cathode and anode. For OWS and FOR-WS, a H-type cell (50 mL of volume for each chamber) was used with an anion exchange membrane (AEM) to separate the cathodic and anodic chambers. For OWS, both two chambers were filled with 40 mL of 1 M KOH. For FOR-WS, the cathodic chamber was filled with 40 mL of 1 M KOH, while the anodic chamber was filled with 40 mL of 1 M KOH & 0.6 M HCHO. For FPFC, a bipolar membrane (BPM) was applied in a H-type cell (50 mL of volume for each chamber), with an aim to separate 40 mL of 0.5 M H_2_SO_4_ in cathodic chamber and 40 mL of 1 M KOH & 0.6 M HCHO in anodic chamber. LSV curves and power-density curves of FPFC were measured at a scan rate of 2 mV s^−1^ without *iR* compensation. Galvanostatic discharge tests were conducted at sequential current densities of 2, 5, 10 and 20 mA cm^−2^, with each step lasting 30 min. To evaluate long-term durability, a smaller H-type cell (10 mL of volume in each chamber) was used for FPFC with 10 mL of electrolytes in each chamber. FPFC was connected with a constant load using an external resistor (1000 Ω). After each cycle, both the cathodic and anodic electrolytes were replaced with fresh solutions.

**Product analysis**

The H_2_ yield rates (*r*) and Faradaic efficiency (*FE*) were calculated as follows:

$$n_{H_{2}}\text{=}\frac{V_{H_{2}}}{\text{22.4}}$$

$$\text{r}\text{=}\frac{n_{H_{2}}}{\text{A}\text{×}\text{t}}$$

$$\text{FE}\text{=}\frac{\text{F×}n_{H_{2}}\text{×}\text{z}}{\text{Q}}\text{×100}\text{\%}$$

$\text{V}_{\text{H}_{\text{2}}}$: Volume of H_2_ produced measured by the water displacement method.

*A*: The effective contact area between the catalyst and the electrolyte is 0.25 cm².

*t*: Electrolysis time is 60 min.

F: Faraday constant (96485 C mol^−1^).

*z*: The number of electrons transferred, where *z*(H₂) = 2.

*Q*: Applied electric charge during electrolysis.

The produced formate and methanol in the electrolyte were quantitatively determined by ^1^H NMR spectroscopy using dimethyl sulfoxide (DMSO) as the internal standard. To prepare the NMR solution, 0.45 mL of electrolyte was sequentially mixed with 0.05 mL of HCl (37%), 0.04 mL of D_2_O and 0.05 mL of DMSO (0.7 M in H_2_O). ^1^H NMR spectra were recorded for the electrolyte before and after the 1 h electrolysis of Ru-Cu NTs@CM in 1 M KOH & 0.6 M HCHO at different potentials from 0 to 0.4 V *vs.* RHE.

The concentration of HCHO in electrolyte was measured by UV-Vis absorption spectroscopy. The chromogenic reagent was prepared by dissolving 7.5 g of acetamide, 150 μL of glacial acetic acid, and 100 μL of acetylacetone in 49.75 mL of deionized water. For the determination of HCHO concentration, 20 μL of electrolyte was acidified with 20 μL of 2 M HCl and diluted 2500-fold with deionized water. Subsequently, 2 mL of the diluted solution was mixed with 2 mL of the chromogenic reagent, and the mixture was heated in an oven at 60 °C for 10 min to ensure complete reaction. After full cooling, the absorbance of the solution was measured at 413 nm. A standard curve was obtained using commercially available HCHO solutions with known concentrations, and the quantitative determination of HCHO was performed based on this standard curve.

The carbon balance of FOR electrolysis was calculated as follows:

$$\text{C}\text{arbon ba}\text{l}\text{ance=}\frac{\text{mol of }\text{produced formate from FOR}}{\text{mol of consumed}\text{ HCHO from FOR}}\text{×10}\text{0\%}$$

Given that the Cannizzaro reaction ($2HCHO+\mathrm{OH}^{-}\to\mathrm{CH}_{3}OH+\mathrm{HCOO}^{-}$) co-occurs, producing methanol and formate, both the consumed HCHO and produced formate in the electrolyte must be substracted. ^1^H NMR spectroscopy measurements were applied to evaluate the amount of methanol and formate. In addition, the quantitative determination of consumed HCHO was measured by UV-Vis absorption spectroscopy. By determining the produced methanol before and after FOR electrolysis, the consumed HCHO and produced formate from Cannizzaro reaction can be easily calculated based on its reaction equation. Then, the consumed HCHO and produced formate from FOR can be obtained.

***In situ* electrochemical Raman spectroscopy measurements**

*In situ* Raman spectroscopy measurements were performed on a Renishaw InVia confocal laser Raman spectrometer with excitation by a 532 nm laser. A commercial one-compartment polytetrafluoroethylene (PTFE) electrochemical cell was used for recording *in situ* Raman spectra, with the self-supported electrode as working electrode, Pt wire as counter electrode, SCE as reference electrode, and 1 M KOH & 0.6 M HCHO as electrolyte. For Cu NWs@CM and Ru-Cu NTs@CM, Raman spectra were collected for every 5 min at a constant potential of 0.8 V *vs.* RHE. For Ru-Cu NTs@CM, Raman spectra were also *in situ* recorded after 5 min of FOR electrolysis at different potentials.

***In situ* differential electrochemical mass spectrometry measurements**

Isotope-labeled online differential electrochemical mass spectrometry (DEMS) measurements were performed using a LingLu instrument (QAS 100, Shanghai) to real-time monitor small molecular volatile substances generated during FOR. Volatile molecules enter the vacuum system through a hydrophobic PTFE membrane, which allows small molecules to pass through while effectively blocking the aqueous solution from entering the vacuum chamber, and is crucial for maintaining the vacuum environment. A three-electrode system was fabricated with Ru-Cu NTs@CM as working electrode, Pt wire as counter electrode, SCE as reference electrode, and 1 M KOH & 0.6 M HCHO as electrolyte. The measurement employed a cyclic protocol comprising a 200 s electrolysis at a constant 0.4 V *vs.* RHE, followed by a 200 s relaxation phase without any applied potential. The relaxation phase was used to remove residual bubbles from the previous cycle. Formal data collection was started after the mass spectrometry signal baseline became stable.

**DFT calculations**

DFT calculations were performed in Vienna Ab-initio Simulation Package (VASP) alongside the Atomic Simulation Environment (ASE). The calculation of exchange and correlation energies utilized the Bayesian Error Estimation Functional with van der Waals correlations (BEEF-vdW) exchange-correlation functional. BEEF-vdW was selected for its proven accuracy in predicting small-molecule adsorption energies on transition-metal surfaces. The inner electrons were described through projector augmented wave (PAW) pseudopotentials, with the plane-wave cutoff energy established at 400 eV. The Brillouin zone sampling was executed *via* a Γ-centered k-point mesh, generated employing the Monkhorst-Pack method. Geometry optimizations proceeded until the force on each atom diminished below the convergence threshold of 0.02 eV Å^−1^, ensuring energy convergence within 10^−6^ eV. For the adsorption energy calculation, it was obtained as:

$$\begin{aligned} E_{\mathrm{ads}}=E_{\mathrm{total}}-E_{\mathrm{surf}}-E_{\mathrm{adsorbate}} \end{aligned}$$

where *E*_total_ is the total energy of molecule absorbate covered on a surface, *E*_surf_ is the energy of a surface, and *E*_adsorbate_ is the energy of molecule in vacuum.

Climbing Image Nudged Elastic Band (CINEB) and dimer methods were employed to investigate the transition states of elemental reactions. And all of the [transition state structures](https://www.sciencedirect.com/topics/chemistry/transition-state-structure) were tested to make sure only one imaginary frequency. The reaction barrier was determined as:

$$\begin{aligned} E_{\mathrm{barrier}}=E_{\mathrm{TS}}-E_{\mathrm{IS}} \end{aligned}$$

where *E*_TS_ denotes the energy of transition state, *E*_IS_ denotes the energy of initial state. The [thermochemistry](https://www.sciencedirect.com/topics/chemistry/thermochemistry) was calculated by the package of VASPKIT.

For FOR, it involves a series of consecutive elementary steps for DFT calculations:

$$*+H_{2}C{(OH)}_{2}\to H_{2}C{{(OH)}_{2}}^{*}$$

$$H_{2}C{{(OH)}_{2}}^{*}+\mathrm{OH}^{-}+e^{-}\to H_{2}C{{(OH)}_{2}}^{*}+\mathrm{OH}^{*}$$

$$H_{2}C{{(OH)}_{2}}^{*}+\mathrm{OH}^{*}\to H_{2}C\mathrm{OOH}^{*}+H_{2}O^{*}$$

$$H_{2}C\mathrm{OOH}^{*}+H_{2}O^{*}\to H_{2}C\mathrm{OOH}^{*}+H_{2}O$$

$$H_{2}C\mathrm{OOH}^{*}\to HC\mathrm{OOH}^{*}+H^{*}$$

$$2H^{*}\to H_{2}$$

$$\mathrm{HC}\mathrm{OOH}^{*}\to HCOOH+*$$

**Thermodynamic calculations and voltage conversions**

(i) Conventional OWS is constructed by coupling cathodic HER and anodic OER.

OWS：$H_{2}O\to H_{2}+1/2O_{2}$

Δ_f_*G*_m_^θ^_(H2O)_ = -237.1 kJ mol^−1^, Δ_f_*G*_m_^θ^_(H2)_ = 0 kJ mol^−1^, Δ_f_*G*_m_^θ^_(O2)_ = 0 kJ mol^−1^

Δ_r_*G*_m_^θ^_OWS_ = Δ_f_*G*_m_^θ^_(H2)_ + 1/2Δ_f_*G*_m_^θ^_(O2)_ − Δ_f_*G*_m_^θ^_(H2O)_ = 237.1 kJ mol^−1^

Δ_r_*G*_m_^θ^_OWS_ is positive, indicating OWS is a nonspontaneous reaction. OWS requires electricity input to trigger the reaction.

Δ_r_*G*_m_^θ^_OWS_ = -*n*F*E*_OWS_^θ^ (*n* = 2, F = 96485 C mol^−1^)

*E*_OWS_^θ^ = *E*_HER_^θ^ – *E*_OER_^θ^ = -Δ_r_*G*_m_^θ^_OWS_/*n*F = -1.229 V

It indicates that electricity input with a theoretical voltage of 1.229 V is indispensable to trigger OWS.

*In acidic media (pH = 0):*

Cathodic HER: $2H^{+}+2e^{-}\to H_{2}$

$$E_{\mathrm{HER}}\left( pH=0 \right)=E_{H^{+}/H_{2}}^{\theta}+\frac{RT}{2F}\ln\frac{[a_{H^{+}}]^{2}}{\left[ a_{H_{2}} \right]}=0 V-0.0591\times pH_{\mathrm{Cathode}}=0 V vs.\mathrm{SHE}$$

Anodic OER: $H_{2}O\to1/2O_{2}+2H^{+}+2e^{-}$

$$E_{\mathrm{OER}}\left( pH=0 \right)=E_{O_{2}{/H}_{2}O}^{\theta}+\frac{RT}{2F}\ln\frac{[a_{H^{+}}]^{2}\left[ a_{O_{2}} \right]^{1/2}}{\left[ a_{H_{2}O} \right]}=1.229 V-0.0591\times pH_{\mathrm{Anode}}=1.229 V vs.\mathrm{SHE}$$

*In alkaline media (pH = 14, pOH = 0):*

Cathodic HER: ${2H}_{2}O+2e^{-}\to H_{2}+2OH^{-}$

$$E_{\mathrm{HER}}\left( pH=14 \right)=E_{H_{2}O/H_{2}}^{\theta}+\frac{RT}{2F}\ln\frac{\left[ a_{H_{2}O} \right]^{2}}{\left[ a_{H_{2}} \right]\left[ a_{\mathrm{OH}^{-}} \right]^{2}}=-0.828 V+0.0591\times pOH_{\mathrm{Cathode}}=-0.828 V vs.\mathrm{SHE}$$

Anodic OER: $2OH^{-}\to1/2O_{2}+H_{2}O+2e^{-}$

$$E_{\mathrm{OER}}\left( pH=14 \right)=E_{O_{2}/OH^{-}}^{\theta}+\frac{RT}{2F}\ln\frac{\left[ a_{O_{2}} \right]^{1/2}\left[ a_{H_{2}O} \right]}{\left[ a_{\mathrm{OH}^{-}} \right]^{2}}=0.401 V+0.0591\times{pOH}_{\mathrm{Anode}}=0.401 V vs.\mathrm{SHE}$$

(ii) Small-molecules oxidation reactions assisted water splitting (SMORs-WS) is constructed by coupling cathodic HER and anodic SMORs.

SMORs-WS: $H_{2}O+\left[ Red \right]\to H_{2}+[Ox]$

([*Red*] represents reduced species, whereas [*Ox*] represents oxidized species)

Δ_r_*G*_m_^θ^_SMORs-WS_ = Δ_f_*G*_m_^θ^_(H2)_ + Δ_f_*G*_m_^θ^_[_*_Ox_*_]_ − Δ_f_*G*_m_^θ^_(H2O)_ − Δ_f_*G*_m_^θ^_[_*_Red_*_]_ = 237.1 kJ mol^−1^ + Δ_f_*G*_m_^θ^_[_*_Ox_*_]_ − Δ_f_*G*_m_^θ^_[_*_Red_*_]_ = Δ_r_*G*_m_^θ^_OWS_ + Δ_f_*G*_m_^θ^_[_*_Ox_*_]_ − Δ_f_*G*_m_^θ^_[_*_Red_*_]_

Δ_r_*G*_m_^θ^_SMORs-WS_ − Δ_r_*G*_m_^θ^_OWS_ = Δ_f_*G*_m_^θ^_[_*_Ox_*_]_ − Δ_f_*G*_m_^θ^_[_*_Red_*_]_

Therefore, if we want to achieve a decreased Δ_r_*G*_m_^θ^_SMORs-WS_ compared to Δ_r_*G*_m_^θ^_OWS_, Δ_f_*G*_m_^θ^_[_*_Ox_*_]_ should be lower than Δ_f_*G*_m_^θ^_[_*_Red_*_]_. For example, formaldehyde oxidation reaction (FOR)-assisted water splitting (FOR-WS) is generally constructed by coupling cathodic HER and anodic FOR in alkaline media.

FOR-WS: $H_{2}O+HCHO\to H_{2}+HCOOH$

Δ_f_*G*_m_^θ^_(H2O)_ = -237.1 kJ mol^−1^, Δ_f_*G*_m_^θ^_(H2)_ = 0 kJ mol^−1^, Δ_f_*G*_m_^θ^_(HCHO)_ = -102.7 kJ mol^−1^, Δ_f_*G*_m_^θ^_(HCOOH)_ = -361.4 kJ mol^−1^

HCHO is the reduced species, whereas HCOOH is the oxidized species. Δ_f_*G*_m_^θ^_(HCOOH)_ is much lower than Δ_f_*G*_m_^θ^_(HCHO)_.

Δ_r_*G*_m_^θ^_FOR-WS_ = Δ_f_*G*_m_^θ^_(H2)_ + Δ_f_*G*_m_^θ^_(HCOOH)_ − Δ_f_*G*_m_^θ^_(H2O)_ − Δ_f_*G*_m_^θ^_(HCHO)_

= -21.6 kJ mol^−1^

Δ_r_*G*_m_^θ^_FOR-WS_ is negative, indicating FOR-WS is a spontaneous reaction.

Δ_r_*G*_m_^θ^_FOR-WS_ = -*n*F*E*_FOR-WS_^θ^ (*n* = 1, F = 96485 C mol^−1^)

*E*_FOR-WS_^θ^ = *E*_HER_^θ^ – *E*_FOR_^θ^ = -Δ_r_*G*_m_^θ^_FOR-WS_/*n*F = 0.224 V

It indicates that FOR-WS can achieve electricity output with a theoretical voltage of 0.224 V, however, electricity input generally still required to reach an appreciable current density (for instance, ≥ 100 mA cm^−2^) due to the existence of overpotentials and *IR* drop.

*In alkaline media (pH = 14, pOH = 0):*

Cathodic HER: $H_{2}O+e^{-}\to{1/2H}_{2}+OH^{-}$

$$E_{\mathrm{HER}}\left( pH=14 \right)=E_{H_{2}O/H_{2}}^{\theta}+\frac{RT}{F}\ln\frac{\left[ a_{H_{2}O} \right]}{\left[ a_{H_{2}} \right]^{1/2}\left[ a_{\mathrm{OH}^{-}} \right]}=-0.828 V+0.0591\times pOH_{\mathrm{Cathode}}=-0.828 V vs.\mathrm{SHE}$$

Anodic FOR: $\mathrm{HCHO}+OH^{-}\to{1/2H}_{2}+HCOOH+e^{-}$

$$E_{\mathrm{FOR}}\left( pH=14 \right)=E_{HCOOH/HCHO}^{\theta}+\frac{RT}{F}\ln\frac{\left[ a_{H_{2}} \right]^{1/2}\left[ a_{\mathrm{HCOOH}} \right]}{\left[ a_{\mathrm{HCHO}} \right]\left[ a_{\mathrm{OH}^{-}} \right]}=E_{HCOOH/HCHO}^{\theta}+0.0591\times{pOH}_{\mathrm{Anode}}=E_{HCOOH/HCHO}^{\theta}$$

Given that *E*_FOR-WS_^θ^ is 0.224 V, $E_{HCOOH/HCHO}^{\theta}$ is calculated as -1.052 V *vs.* SHE.

(iii) Hybrid alkaline-acidic FPFC is constructed by coupling acidic HER at cathode and alkaline FOR at anode.

FPFC: $H^{+}+\mathrm{OH}^{-}+HCHO\to H_{2}+HCOOH$

Δ_f_*G*_m_^θ^_(H+)_ = 0 kJ mol^−1^, Δ_f_*G*_m_^θ^_(OH-)_ = -157.2 kJ mol^−1^, Δ_f_*G*_m_^θ^_(H2)_ = 0 kJ mol^−1^, Δ_f_*G*_m_^θ^_(HCHO)_ = -102.7 kJ mol^−1^, Δ_f_*G*_m_^θ^_(HCOOH)_ = -361.4 kJ mol^−1^

Δ_r_*G*_m_^θ^_FPFC_ = Δ_f_*G*_m_^θ^_(H2)_ + Δ_f_*G*_m_^θ^_(HCOOH)_ − Δ_f_*G*_m_^θ^_(H+)_ − Δ_f_*G*_m_^θ^_(OH-)_ − Δ_f_*G*_m_^θ^_(HCHO)_

= -101.5 kJ mol^−1^

Compared with Δ_r_*G*_m_^θ^_FOR-WS_ (-21.6 kJ mol^−1^), Δ_r_*G*_m_^θ^_FPFC_ (-101.5 kJ mol^−1^) is decreased by 79.9 kJ mol^−1^, demonstrating FPFC shows superior thermodynamic property to FOR-WS. The reduced Δ_r_*G*_m_^θ^ value (-79.9 kJ mol^−1^) is derived from the acid-alkali neutralization reaction (AANR) energy.

AANR: $H^{+}+\mathrm{OH}^{-}\to H_{2}O$

Δ_f_*G*_m_^θ^_(H+)_ = 0 kJ mol^−1^, Δ_f_*G*_m_^θ^_(OH-)_ = -157.2 kJ mol^−1^, Δ_f_*G*_m_^θ^_(H2O)_ = -237.1 kJ mol^−1^

Δ_r_*G*_m_^θ^_AANR_ = Δ_f_*G*_m_^θ^_(H2O)_ − Δ_f_*G*_m_^θ^_(H+)_ − Δ_f_*G*_m_^θ^_(OH-)_ = -79.9 kJ mol^−1^

FPFC can be regarded as the combination of FOR-WS and AANR to further reap the Δ_r_*G*_m_^θ^_AANR_ to be electrochemical neutralization energy (ENE). In that case, Δ_r_*G*_m_^θ^_FPFC_ is much more negative than Δ_r_*G*_m_^θ^_FOR-WS_, indicating that FPFC is a thermodynamically more spontaneous reaction.

Δ_r_*G*_m_^θ^_FPFC_ = -*n*F*E*_FPFC_^θ^ (*n* = 1, F = 96485 C mol^−1^)

*E*_FPFC_^θ^ = *E*_Acidic HER_^θ^ – *E*_Alkaline FOR_^θ^ = -Δ_r_*G*_m_^θ^_FPFC_/*n*F = 1.052 V

The result reveals that FPFC can achieve an enhanced theoretical voltage output of 1.052 V with ENE assistance.

*In acidic media (pH = 0) at cathode:*

Cathodic HER: $H^{+}+e^{-}\to{1/2H}_{2}$

$$E_{\mathrm{HER}}\left( pH=0 \right)=E_{H^{+}/H_{2}}^{\theta}+\frac{RT}{F}\ln\frac{[a_{H^{+}}]}{\left[ a_{H_{2}} \right]^{1/2}}=0 V-0.0591\times pH_{\mathrm{Cathode}}=0 V vs.\mathrm{SHE}$$

*In alkaline media (pH = 14, pOH = 0) at anode:*

Anodic FOR: $\mathrm{HCHO}+OH^{-}\to{1/2H}_{2}+HCOOH+e^{-}$

$$E_{\mathrm{FOR}}\left( pH=14 \right)=E_{HCOOH/HCHO}^{\theta}+\frac{RT}{F}\ln\frac{\left[ a_{H_{2}} \right]^{1/2}\left[ a_{\mathrm{HCOOH}} \right]}{\left[ a_{\mathrm{HCHO}} \right]\left[ a_{\mathrm{OH}^{-}} \right]}=E_{HCOOH/HCHO}^{\theta}+0.0591\times{pOH}_{\mathrm{Anode}}=-1.052 V vs.\mathrm{SHE}$$

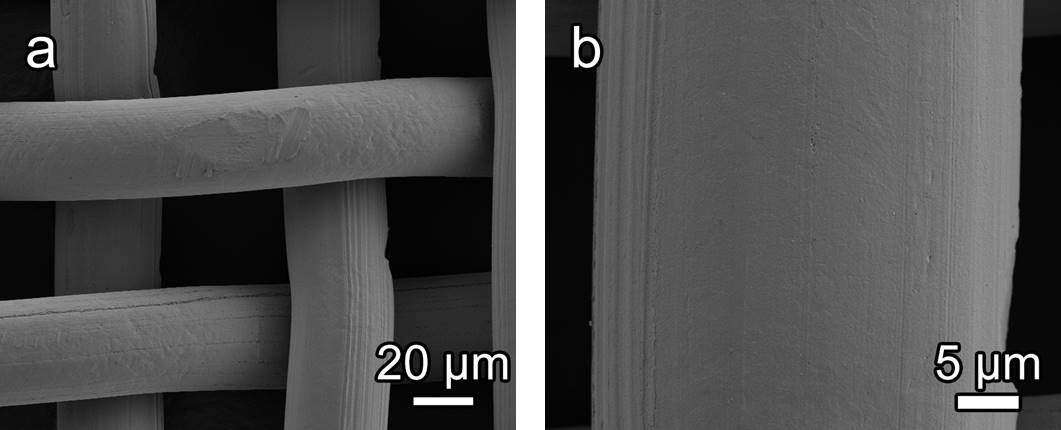


**Figure S1.** SEM images of Cu mesh with smooth surface.


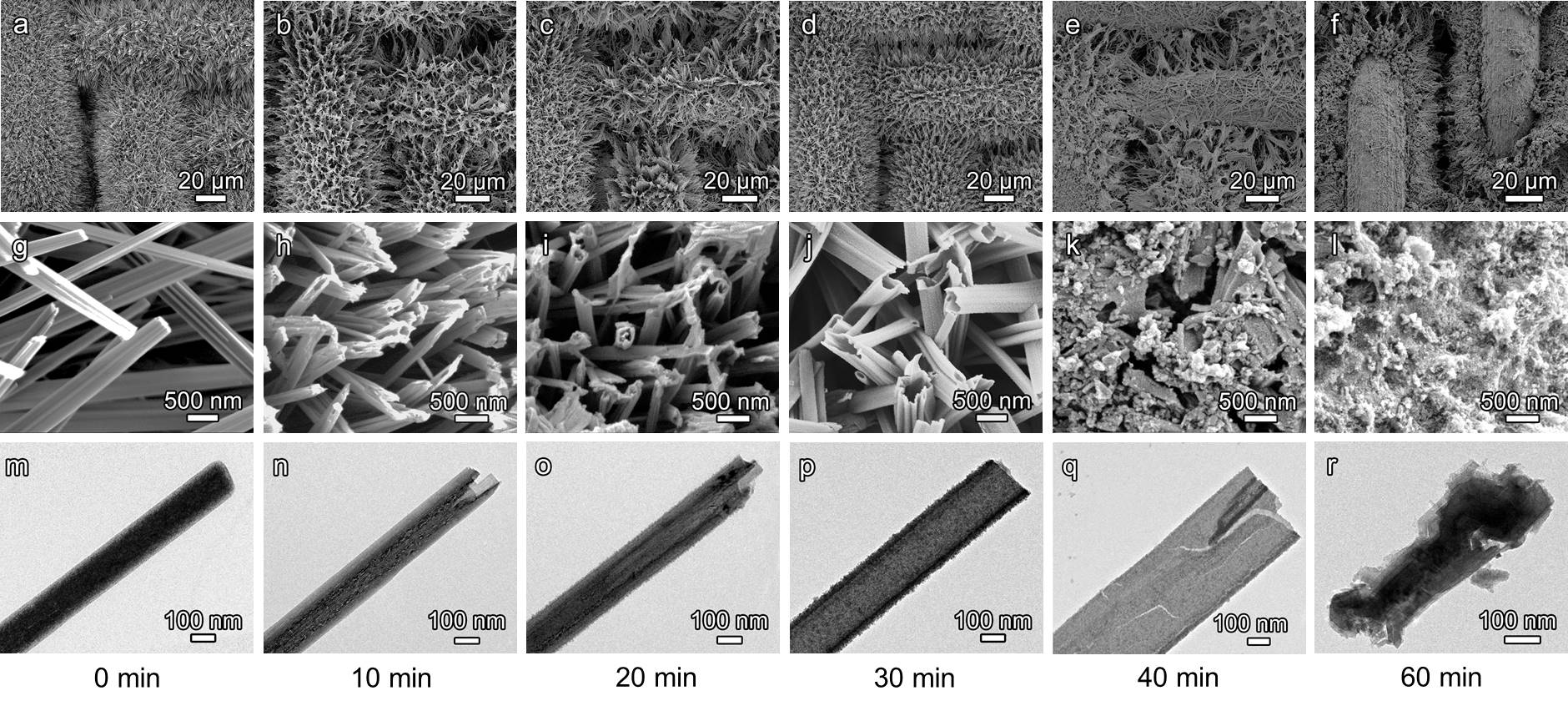


**Figure S2.** The morphological evolution induced by ions exchange process. (a − l) SEM and (m − r) TEM images of samples after immersing Cu(OH)_2_ NWs@CM into 5 mM RuCl_3_ solution for different times (0 to 60 min).

With the proceeding of ions exchange between Ru^3+^ and Cu^2+^, the solid structured Cu(OH)_2_ NWs is gradually converted into the hollow structured Ru-Cu(OH)*_x_* NTs from 0 to 30 min. However, further increasing the exchange time to 40 min or even to 60 min will lead to the fracture and collapse of the samples.


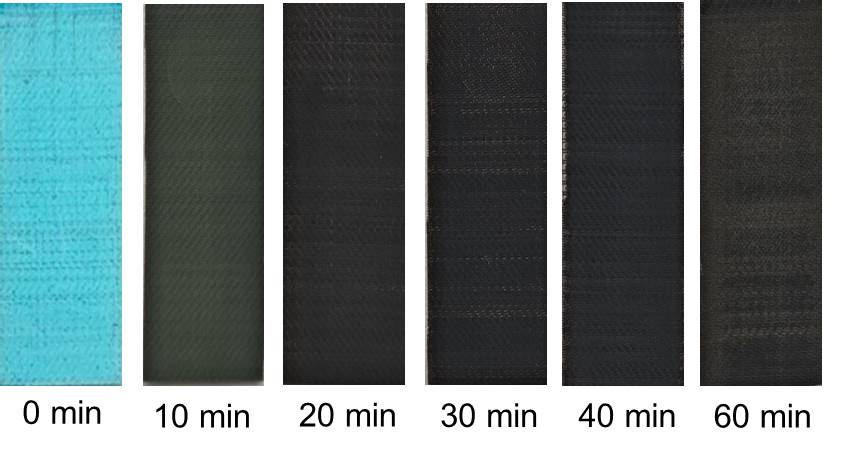


**Figure S3.** Digital photos of samples after immersing Cu(OH)_2_ NWs@CM into 5 mM RuCl_3_ solution for different times (0 to 60 min).

**Figure S4.** XRD patterns of samples after immersing Cu(OH)_2_ NWs@CM into 5 mM RuCl_3_ solution for different times (0 to 60 min).

With the proceeding of ions exchange between Ru^3+^ and Cu^2+^, the intensities of Cu(OH)_2_ XRD feature peaks are decreasing. Three new peaks are gradually appeared at 32.8^o^, 36.6^o^ and 40^o^, which should be indexed to the formation of Ru(OH)_3_.


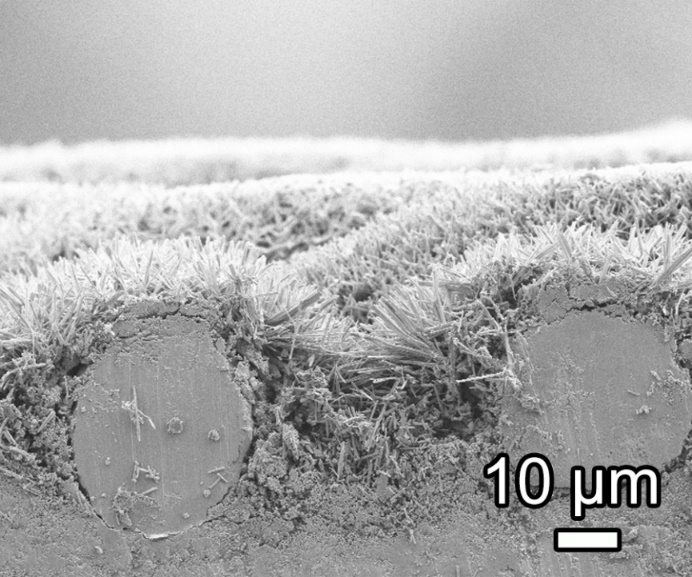


**Figure S5.** The side-view SEM image of Ru-Cu NTs@CM.

**Figure S6.** SEM-EDX spectra of Cu(OH)_2_ NWs@CM, Ru-Cu(OH)*_x_* NTs@CM, Ru-CuO NTs@CM and Ru-Cu NTs@CM.

**Figure S7.** XRD patterns of Cu(OH)_2_ NWs@CM, Ru-Cu(OH)*_x_* NTs@CM, Ru-CuO NTs@CM and Ru-Cu NTs@CM.


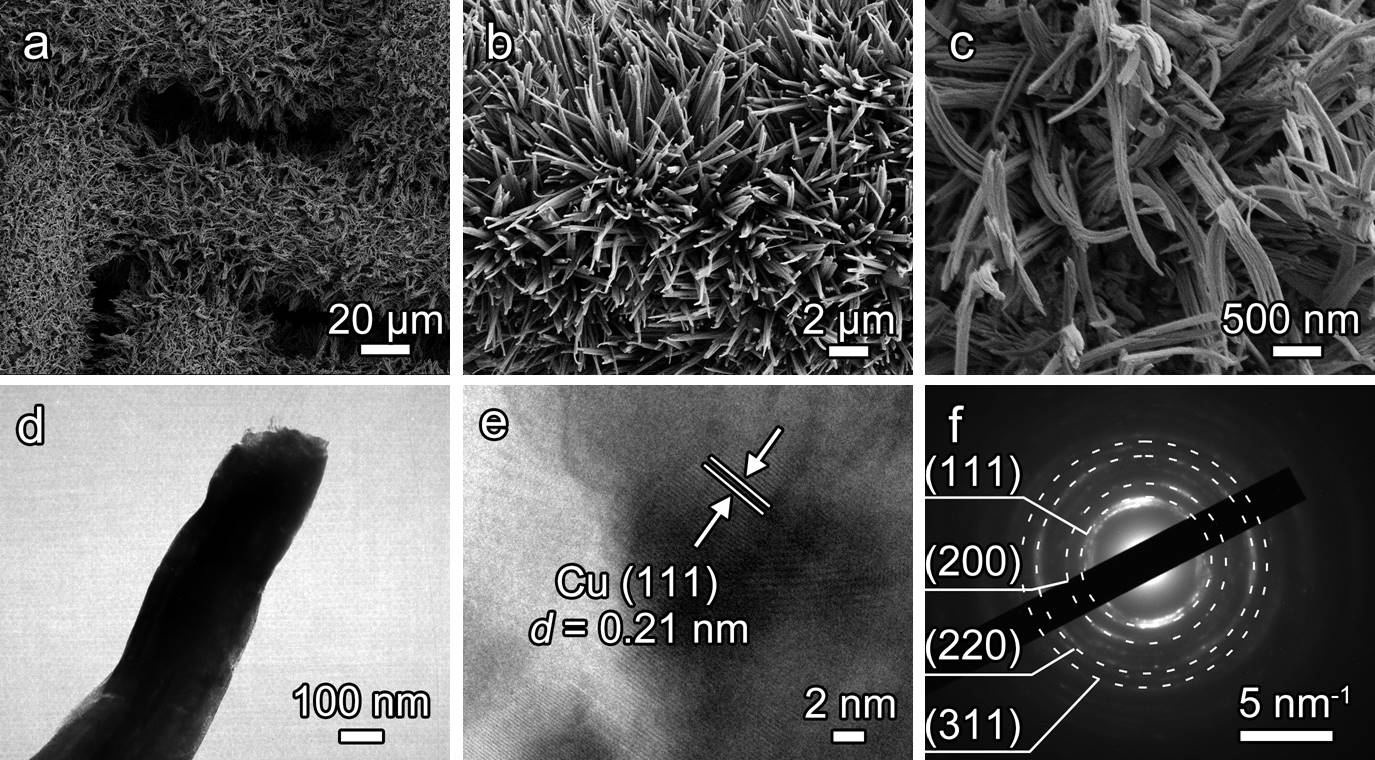


**Figure S8.** (a − c) SEM images, (d) TEM image, (e) HRTEM image and (f) SAED pattern of Cu NWs@CM.


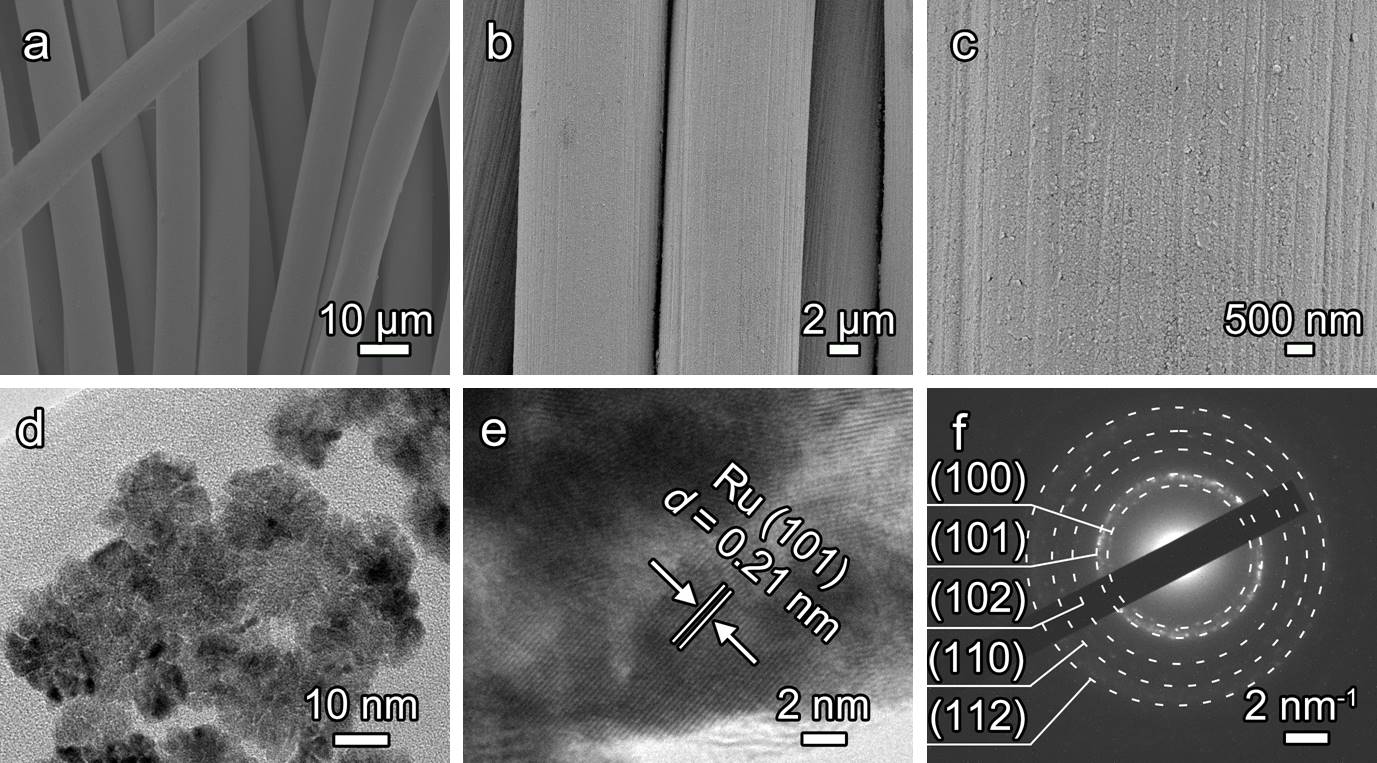


**Figure S9.** (a − c) SEM images, (d) TEM image, (e) HRTEM image and (f) SAED pattern of Ru NPs@CC.

**Figure S10.** (a) FT-EXAFS, (b) k space EXAFS and (c) inversed FT-EXAFS fitting curves of Ru-Cu NTs@CM.

**Figure S11.** (a) FT-EXAFS, (b) k space EXAFS and (c) inversed FT-EXAFS fitting curves of Cu NWs@CM.

**Figure S12.** WT-EXAFS spectra of (a) Ru-Cu NTs@CM (b) Cu NWs@CM.

**Figure S13.** (a − f) SEM images of (a) carbon cloth, (b) Ru NPs@CC-2 min, (c) Ru NPs@CC-4 min, (d) Ru NPs@CC-6 min, (e) Ru NPs@CC-8 min and (f) Ru NPs@CC-10 min. (g) XRD patterns, (h) SEM-EDX spectra and (i) LSV curves in 0.5 M H_2_SO_4_ of the samples indicated in the figures.

**Figure S14.** LSV curves of Cu mesh and carbon cloth in 0.5 M H_2_SO_4_.

**Figure S15.** Nyquist plots of Ru-Cu NTs@CM, Ru NPs@CC and Cu NWs@CM in 0.5 M H_2_SO_4_ at the overpotential of 50 mV.

**Figure S16.** CV curves of (a) Ru-Cu NTs@CM, (b) Cu NWs@CM and (c) Ru NPs@CC within the non-Faradaic voltage region at different scan rates in 0.5 M H_2_SO_4_. (d) The capacitive current as a function of scan rate for the samples (at 0.1 V *vs.* RHE for Ru-Cu NTs@CM and Cu NWs@CM, at 0.5 V *vs.* RHE for Ru NPs@CC).

**Figure S17.** ECSA-normalized LSV curves of Ru-Cu NTs@CM, Ru NPs@CC and Cu NWs@CM for HER in 0.5 M H_2_SO_4_.

The specific capacitance (40 μF cm^−2^) is generally used for the calculation of the ECSA based on the equation: $ECSA= \frac{C_{\mathrm{dl}}}{40 \mu F \mathrm{cm}^{-2} {\mathrm{cm}_{\mathrm{ECSA}}}^{-2}}$

For Ru-Cu NTs@CM in 0.5 M H_2_SO_4_, *ECSA*_Ru-Cu NTs@CM_ = 607.5 cm_ECSA_^2^

For Ru NPs@CC in 0.5 M H_2_SO_4_, *ECSA*_Ru NPs@CC_ = 517.5 cm_ECSA_^2^

For Cu NWs@CM in 0.5 M H_2_SO_4_, *ECSA*_Cu NWs@CM_ = 250 cm_ECSA_^2^

**Figure S18.** (a) XRD pattern and (b) SEM image of Ru-Cu NTs@CM after long-term HER electrolysis in 0.5 M H_2_SO_4_.

**Figure S19.** (a) LSV curves and (b) Tafel plots of electrocatalysts for HER in 1 M KOH.

**Figure S20.** CV curves of (a) Ru-Cu NTs@CM, (b) Cu NWs@CM and (c) Ru NPs@CC within the non-Faradaic voltage region at different scan rates in 1 M KOH. (d) The capacitive current as a function of scan rate for the samples (at 0.1 V *vs.* RHE for Ru-Cu NTs@CM and Cu NWs@CM, at 0.5 V *vs.* RHE for Ru NPs@CC).

**Figure S21.** ECSA-normalized LSV curves of Ru-Cu NTs@CM, Ru NPs@CC and Cu NWs@CM for HER in 1 M KOH.

The specific capacitance (40 μF cm^−2^) is generally used for the calculation of the ECSA based on the equation: $ECSA= \frac{C_{\mathrm{dl}}}{40 \mu F \mathrm{cm}^{-2} {\mathrm{cm}_{\mathrm{ECSA}}}^{-2}}$

For Ru-Cu NTs@CM in 1 M KOH, *ECSA*_Ru-Cu NTs@CM_ = 962.5 cm_ECSA_^2^

For Ru NPs@CC in 1 M KOH, *ECSA*_Ru NPs@CC_ = 877.5 cm_ECSA_^2^

For Cu NWs@CM in 1 M KOH, *ECSA*_Cu NWs@CM_ = 665 cm_ECSA_^2^

**Figure S22.** (a) LSV curves of Ru-Cu NTs@CM before and after 3000 CV cycles under the potential window of -0.1 – 0.1 V *vs.* RHE in 1 M KOH. The inset in (a) presents the long-term electrolysis curve of Ru-Cu NTs@CM under the overpotential of 50 mV in 1 M KOH. (b) XRD pattern and (c) SEM image of Ru-Cu NTs@CM after long-term HER electrolysis in 1 M KOH.

**Figure S23.** LSV curves of Ru-Cu NTs@CM in 1 M KOH containing different concentrations of HCHO from 0 to 0.8 M.

**Figure S24.** (a) Electrolysis curves of Ru-Cu NTs@CM under the potential of 0.3 V *vs.* RHE in 1 M KOH containing ultralow concentrations of HCHO from 5 to 25 mM. (b) The corresponding calibration curve.

**Figure S25.** LSV curves of Cu NWs@CM in 1 M KOH and 1 M KOH & 0.6 M HCHO.

In 1 M KOH, two oxidation peaks at 0.56 and 0.91 V *vs.* RHE are attributable to the oxidation processes of Cu^0^ to Cu^+^ and Cu^0/+^ to Cu^2+^, respectively. Typical, the first Cu oxidation process in alkaline media can be divided into two steps:

(i) $Cu+\mathrm{OH}^{-}\to Cu(OH)_{\mathrm{ad}}+e^{-}$

(ii) $2Cu(OH)_{\mathrm{ad}}\to\mathrm{Cu}_{2}O+H_{2}O$

The first step can initiate on Cu surface at a low potential (< -0.3 V *vs.* RHE). The second step, which requires a high coverage of OH^*^, is not exclusive on Cu surface and can proceed internally. The FOR current density is influenced above the onset potential of the first Cu oxidation process. First, the high coverage of OH^*^ may hinder the adsorption of HCHO-related species on Cu surface, although FOR requires the participation of OH^*^. Second, the inferior conductivity of Cu_2_O layer restricts the rapid electron transfer.

**Figure S26.** LSV curves of Ru NPs@CC in 1 M KOH and 1 M KOH & 0.6 M HCHO.

**Figure S27.** ECSA-normalized LSV curves of Ru-Cu NTs@CM, Ru NPs@CC and Cu NWs@CM for FOR in 1 M KOH & 0.6 M HCHO.

As non-Faradaic voltage region is hardly found for Ru-Cu NTs@CM and Cu NWs@CM in 1 M KOH & 0.6 M HCHO, ECSA values of the electrocatalysts in 1 M KOH were used for FOR normalized LSV curves.


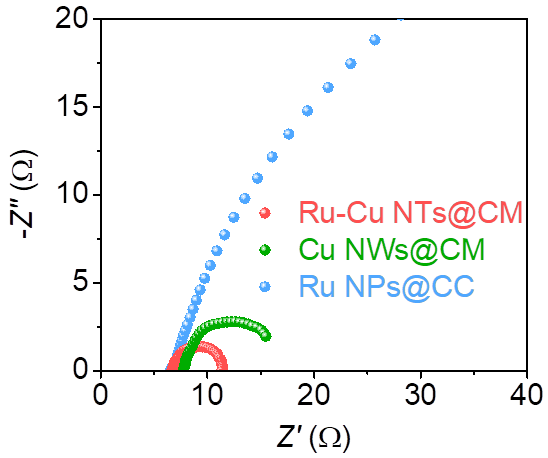


**Figure S28.** Nyquist plots of Ru-Cu NTs@CM, Ru NPs@CC and Cu NWs@CM in 1 M KOH & 0.6 M HCHO at the potential of 0.1 V *vs.* RHE.

**Figure S29.** Electrolysis curves of (a) Ru-Cu NTs@CM, (b) Cu NWs@CM and (c) Ru NPs@CC under different potentials from 0 to 0.4 V *vs.* RHE in 1 M KOH & 0.6 M HCHO.


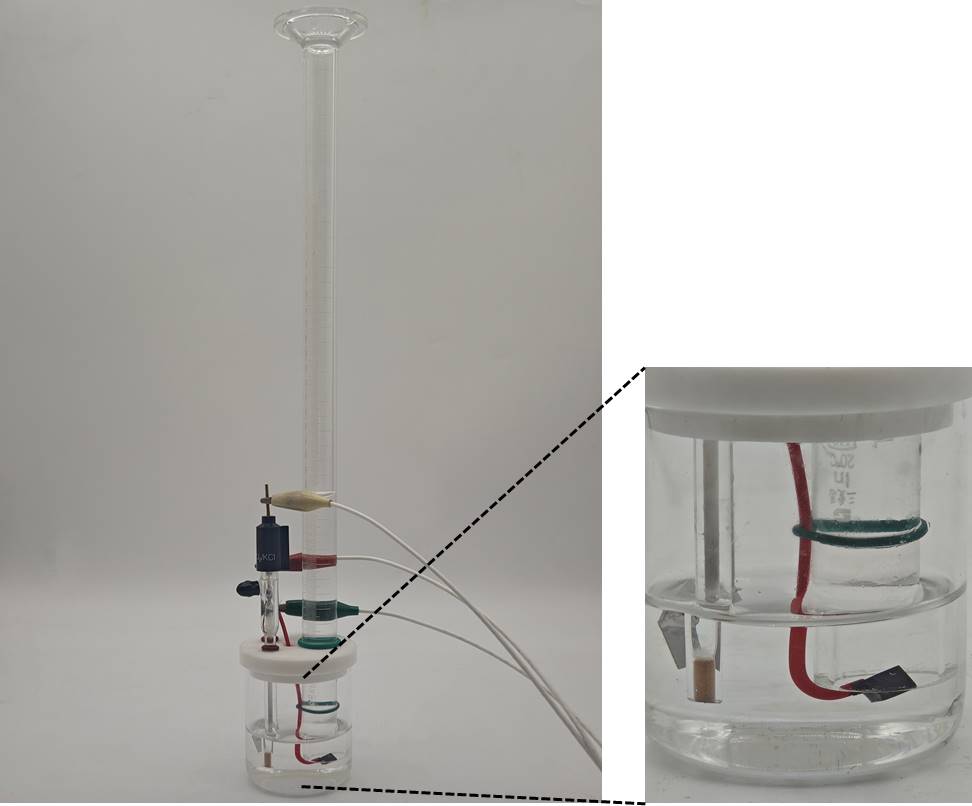


**Figure S30.** Digital photo of the H_2_ collection setup using the water displacement method.


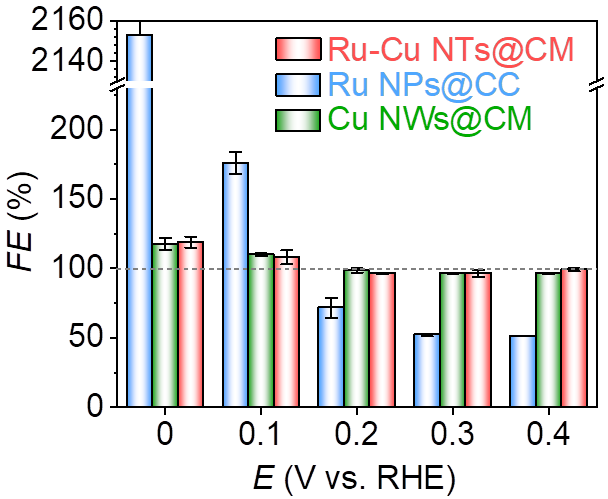


**Figure S31.** Calculated FOR *FEs* for Ru-Cu NTs@CM, Cu NWs@CM and Ru NPs@CC at different potentials from 0 to 0.4 V *vs.* RHE.

**Figure S32.** (a) Volume plots of H_2_ produced *via* the non-Faradaic process (without applied potential) in 1 M KOH & 0.6 M HCHO. (b) H_2_ yield rates of non-Faradaic reaction on Ru-Cu NTs@CM, Cu NWs@CM and Ru NPs@CC.

**Figure S33.** FOR *FEs* of Ru-Cu NTs@CM after electrolysis at 0.4 V *vs.* RHE in 1 M KOH & 0.6 M HCHO for different durations.

**Figure S34.** Comparison of the experimental measured H_2_ amounts and the theoretical H_2_ amounts calculated from the passed charge during FOR electrolysis of Ru-Cu NTs@CM at 0.4 V *vs.* RHE in 1 M KOH & 0.6 M HCHO.

**Figure S35.** (a) ^1^H NMR spectra of the electrolyte before and after the 1 h electrolysis of Ru-Cu NTs@CM in 1 M KOH & 0.6 M HCHO at different potentials from 0 to 0.4 V *vs.* RHE. (b) Molar amounts of HCOOH and CH_3_OH before and after the electrolysis, derived from the ^1^H NMR spectra. (c) Contribution of formate production from FOR and Cannizzaro reaction within the potential range of 0.2 – 0.4 V *vs.* RHE.

There are three pathways for formate production: (i) Cannizzaro reaction in the electrolyte; (ii) electrocatalytic FOR on the catalyst’s surface; (iii) non-Faradaic process on the catalyst’s surface. Among them, only Cannizzaro reaction produces methanol, so it is easy to calculate its contribution based on ^1^H NMR results in Figures S35a and S35b. In contrast, electrocatalytic FOR and non-Faradaic process both produce formate and H_2_, and that is the reason why the calculated *FE* is over 100% at the lower potentials (0 or 0.1 V *vs.* RHE). Notably, their reaction rates are both potential-dependent. When the applied potential is beyond 0.2 V *vs.* RHE, the contribution from non-Faradaic process becomes negligible. Therefore, the chemical (Cannizzaro reaction) and electrochemical (electrocatalytic FOR) contributions are compared within the potential range of 0.2 – 0.4 V *vs.* RHE in Figure S35c. The electrochemical contribution becomes more significant as the applied potential is increased, which is consistent with its faster kinetics at higher potentials.

**Figure S36.** (a) UV-Vis absorption spectra of stained HCHO solution with various concentrations. (b) The corresponding calibration curve used for the estimation of HCHO concentration. (c) UV-Vis absorption spectra of the stained electrolytes after the electrolysis of Ru-Cu NTs@CM in 1 M KOH & 0.6 M HCHO at different potentials for 1 h. (d) Carbon balances of FOR at different potentials.

**Figure S37.** Electrolysis curves of Ru-Cu NTs@CM at 0.3 V *vs.* RHE in 1 M KOH & 0.6 M HCHO for 10 cycles. The electrolyte was refreashed after each cycling of 1 h electrolysis.


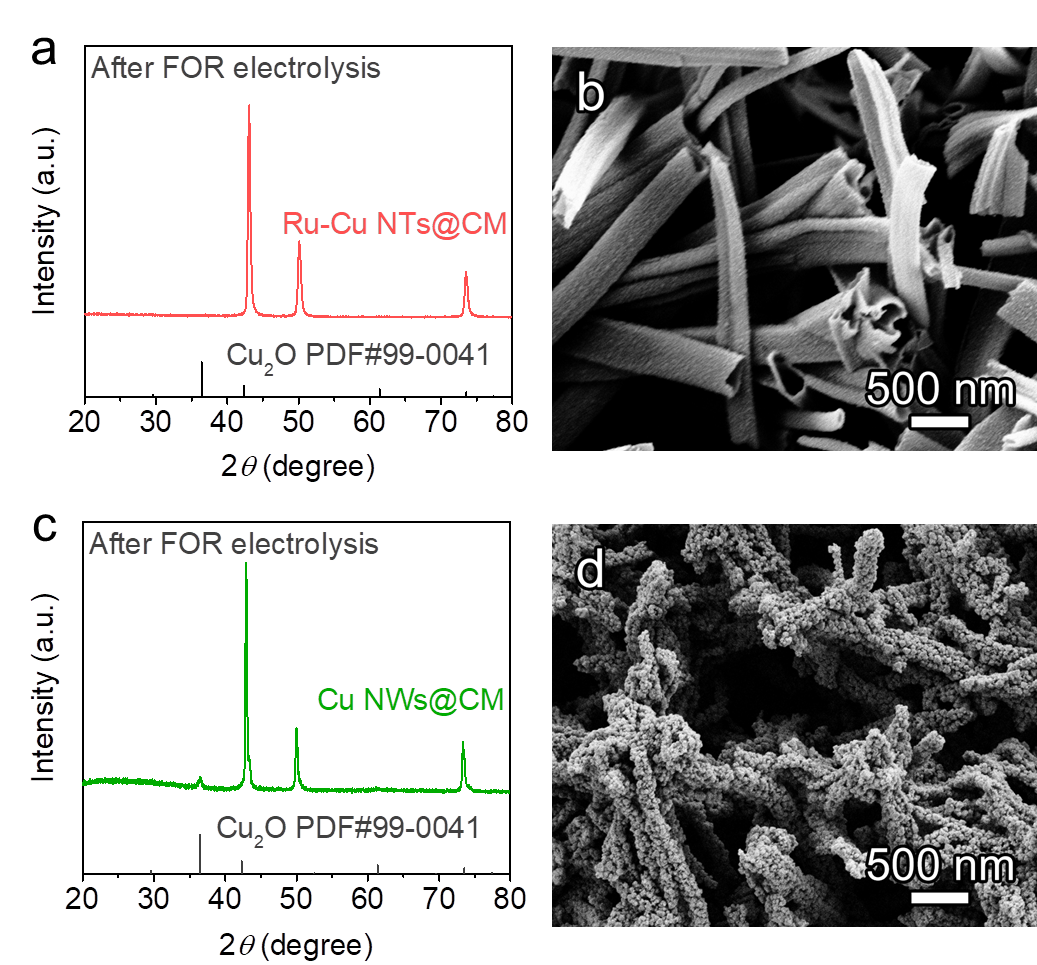


**Figure S38.** (a, c) XRD patterns and (b, d) SEM images of (a, b) Ru-Cu NTs@CM and (c, d) Cu NWs@CM after the FOR electrolysis at 0.75 V *vs.* RHE for 10 h in 1 M KOH & 0.6 M HCHO.

**Figure S39.** Electrolysis curve of Ru-Cu NTs@CM at 0.3 V *vs.* RHE in 1 M KOH with the continuous addition of 0.6 M HCOOH, 0.6 M CH_3_OH and 0.6 M HCHO.

**Figure S40.** GC chromatograms of standard H_2_ and gaseous products from FOR in 1 M KOH & 0.6 M HCHO and HER in 0.5 M H_2_SO_4_.

**Figure S41.** (a) LSV curves of Cu NWs@CM and Ru-Cu NTs@CM in 1 M KOH. (b) XRD patterns of Cu_2_O NWs@CM and Ru-Cu_2_O NTs@CM recorded by the electrolysis of Cu NWs@CM and Ru-Cu NTs@CM at 0.75 V *vs.* RHE in 1 M KOH for 1 min.

In 1 M KOH, LSV curve of Ru-Cu NTs@CM also exhibits two typical Cu oxidation peaks, indicating it can be oxidized at high potentials (> 0.5 V *vs.* RHE) without the presence of HCHO. Compared to Cu NWs@CM, Ru-Cu NTs@CM exhibits the much lower peak current density and the positive shift in the first anodic peak. The results are consistent with the moderated OH^-^ adsorption affinity for Ru-Cu NTs@CM after Ru-doping. After experiencing the electrolysis at 0.75 V *vs.* RHE in 1 M KOH for only 1 min, XRD patterns of the resultant samples (denoted as Cu_2_O NWs@CM and Ru-Cu_2_O NTs@CM) show the existence of Cu_2_O feature peaks.


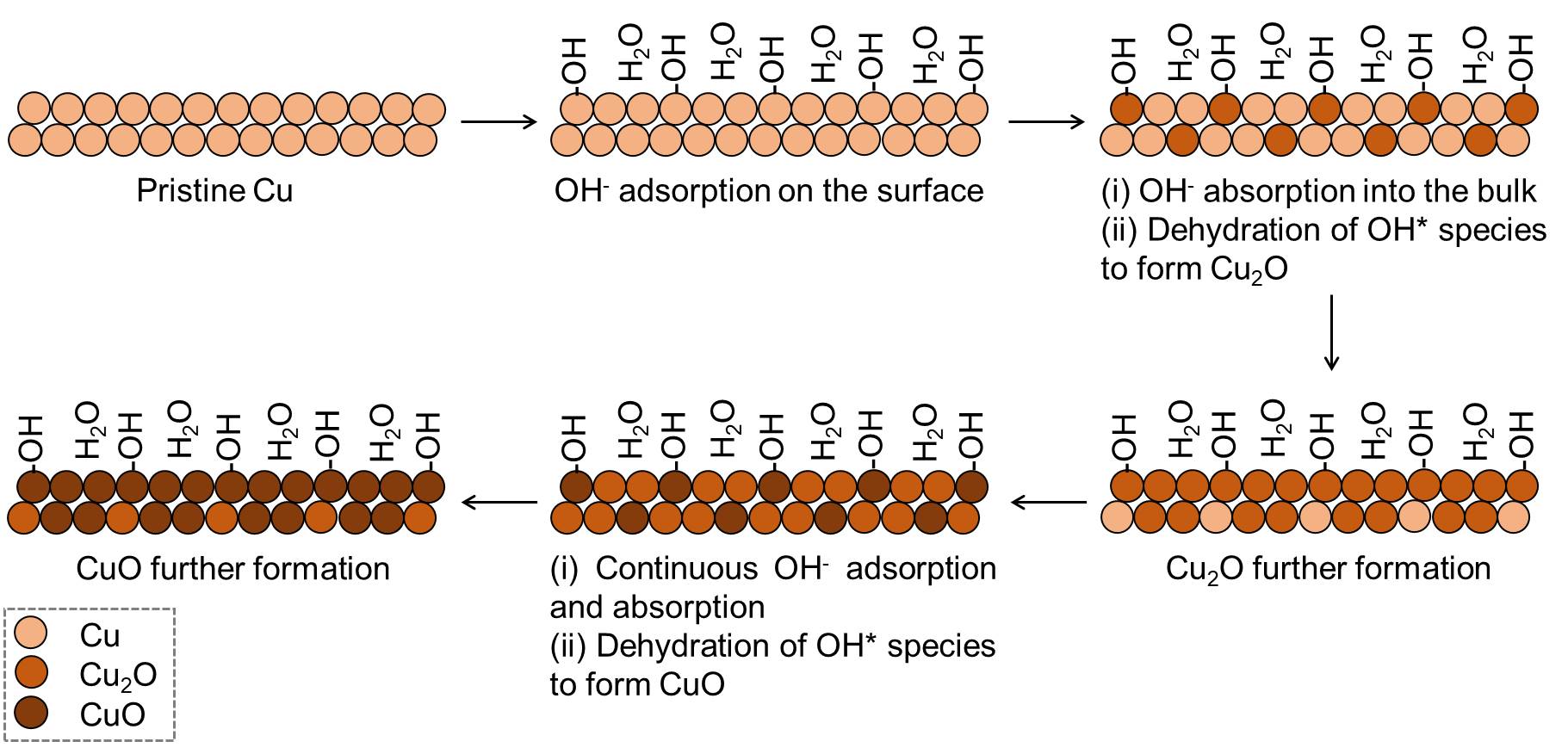


**Figure S42.** Schematic illustration of electrooxidation of Cu in alkaline media. The arrow direction represents the positively shift of potential.


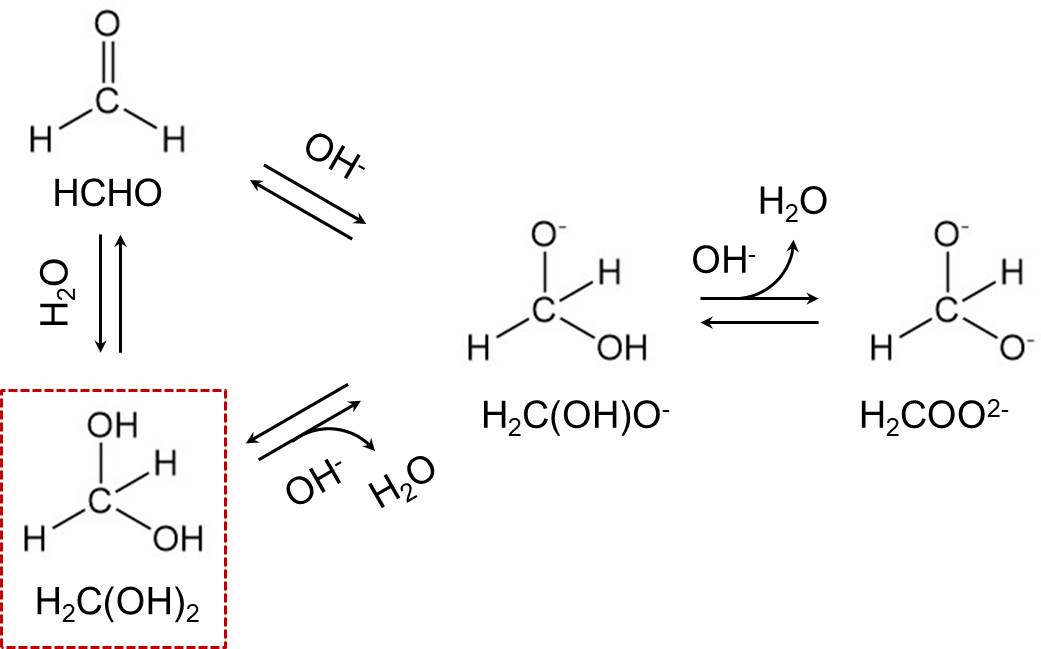


**Figure S43.** The reactions balance of HCHO in alkaline media.


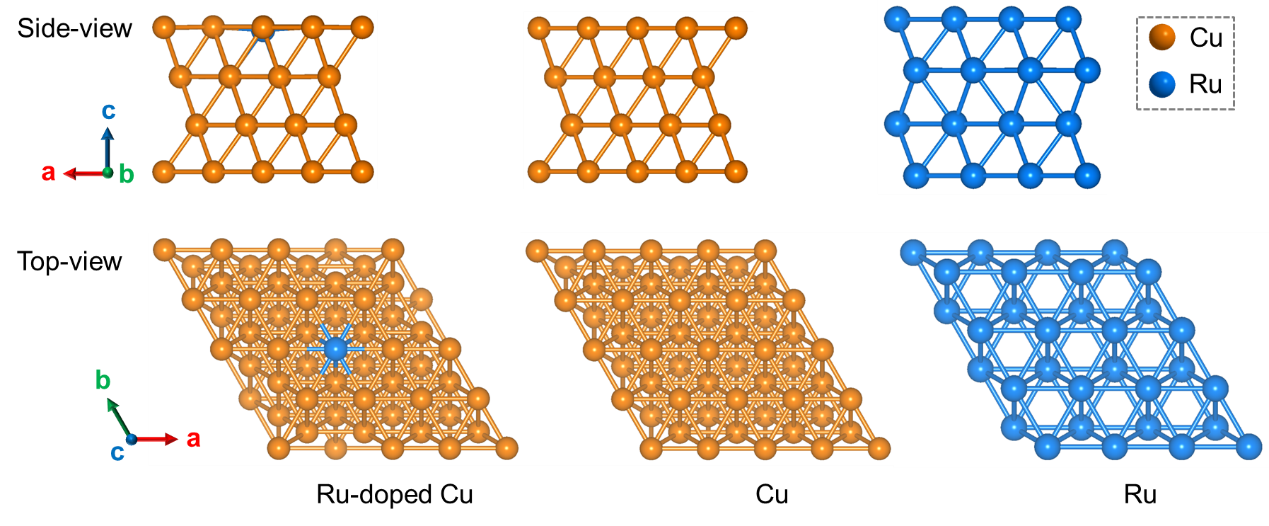


**Figure S44.** Side and top views of schematic models for Ru-doped Cu, Cu and Ru.


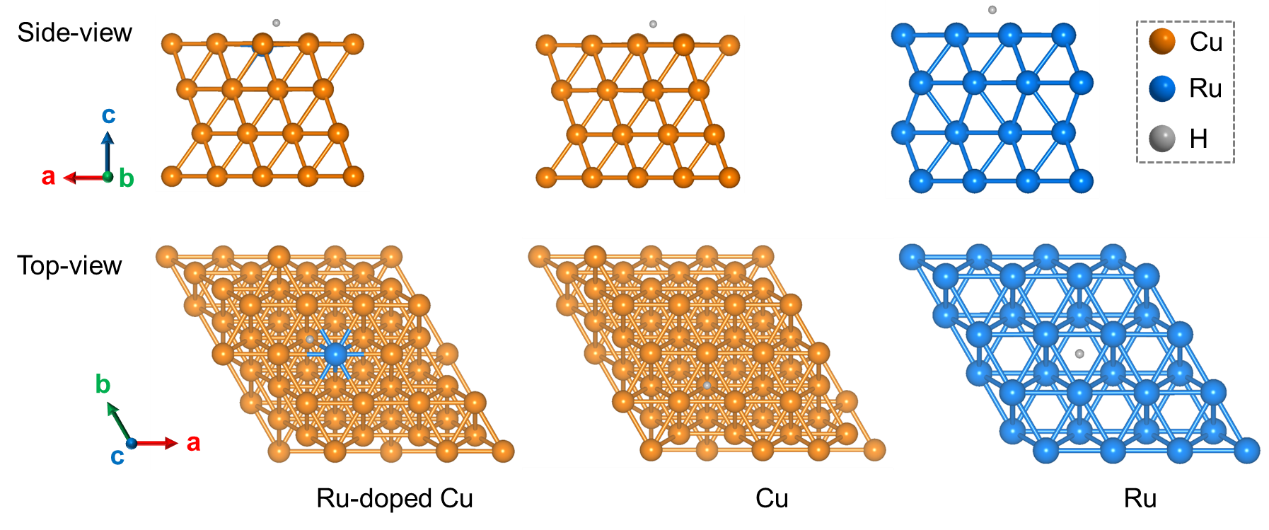


**Figure S45.** Side and top views of schematic models for Ru-doped Cu, Cu and Ru with H^*^ at their optimal catalytic active sites.


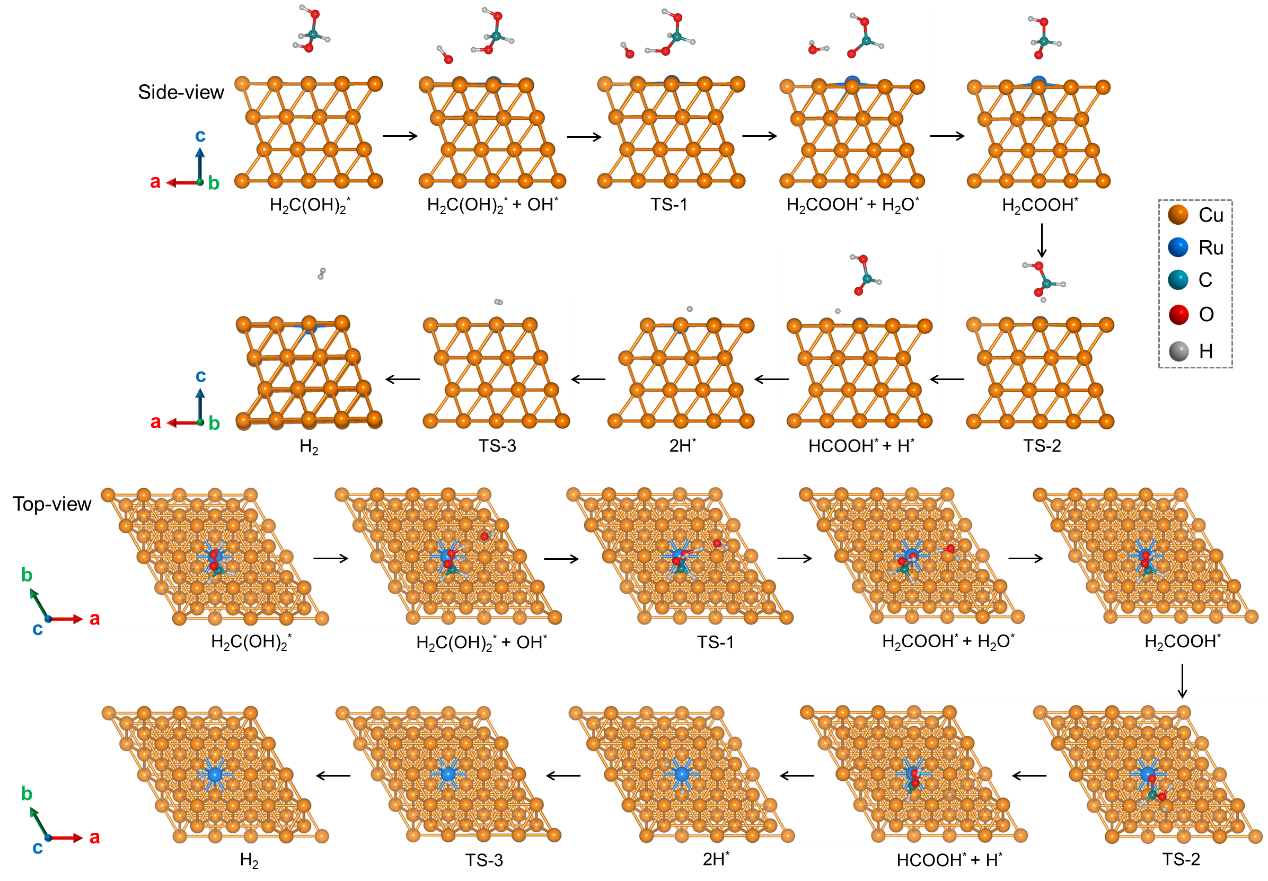


**Figure S46.** Side and top views of schematic illustration of the FOR pathway on Ru-doped Cu.


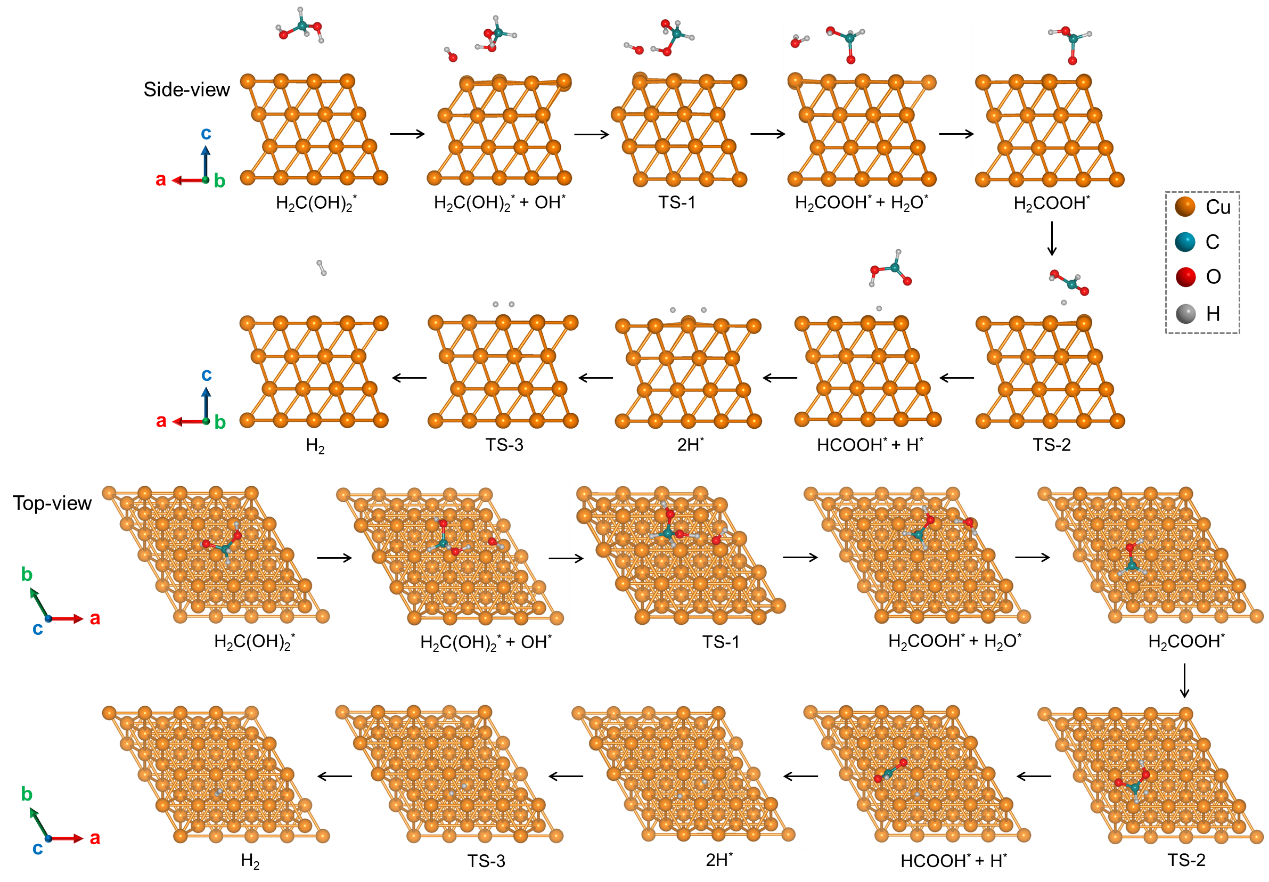


**Figure S47.** Side and top views of schematic illustration of the FOR pathway on Cu.


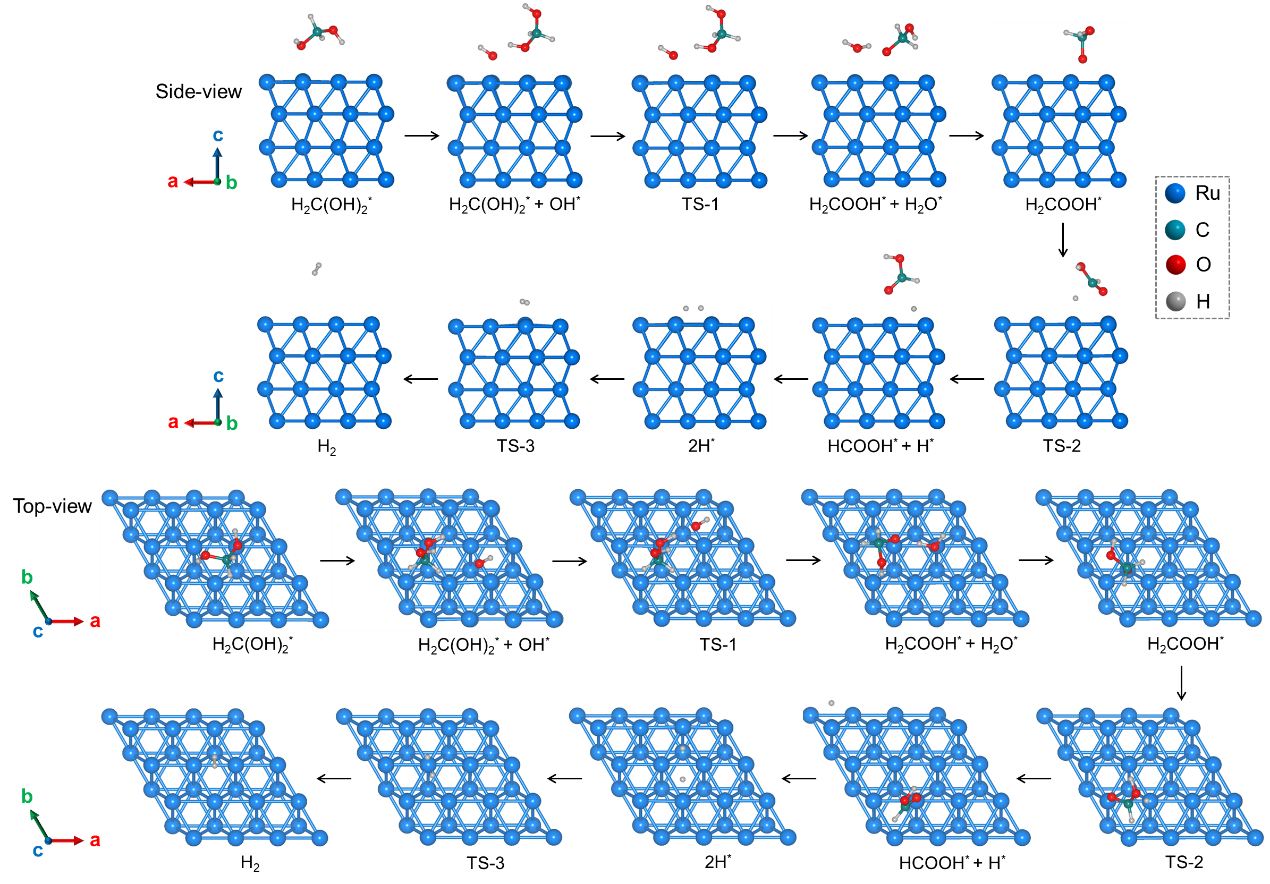


**Figure S48.** Side and top views of schematic illustration of the FOR pathway on Ru.

**Figure S49.** OCVs of the Ru-Cu NTs@CM||Ru-Cu NTs@CM cell recorded under Ar, air and O_2_ atmospheres.


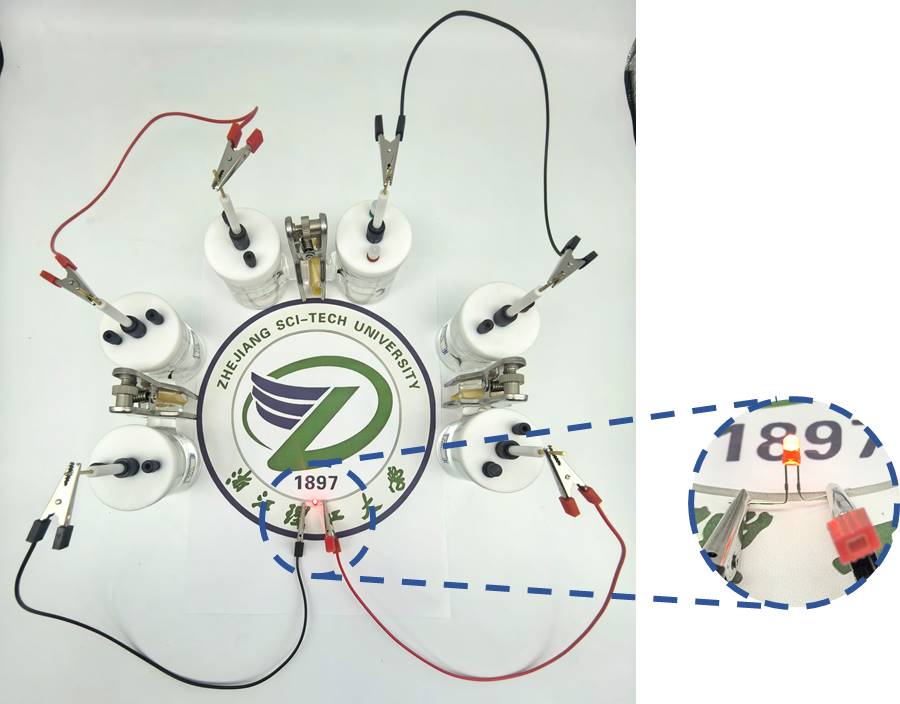


**Figure S50.** Digital image of a red light-emitting diode lighted by three Ru-Cu NTs@CM||Ru-Cu NTs@CM cells connected in series.


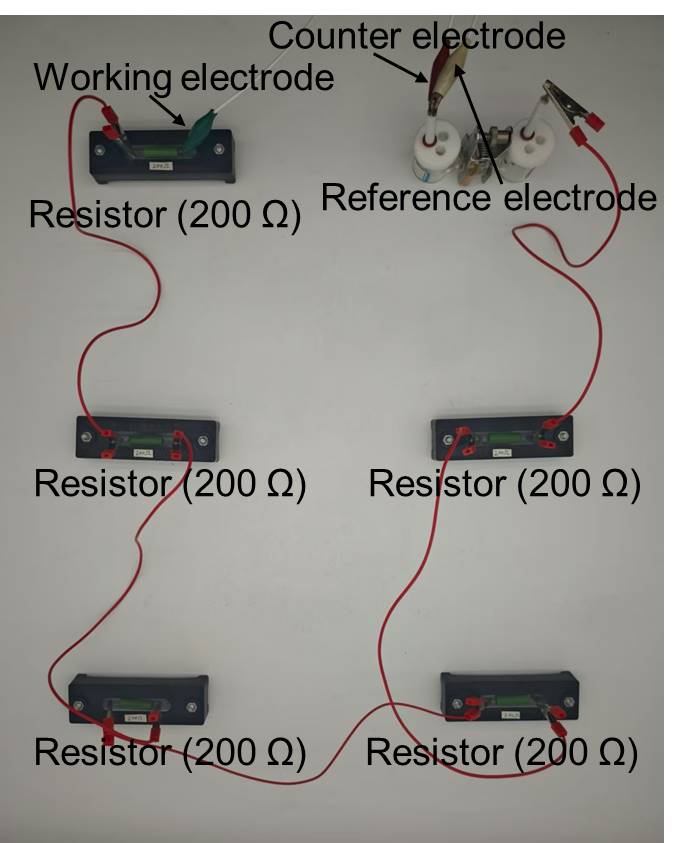


**Figure S51.** Digital image of the Ru-Cu NTs@CM||Ru-Cu NTs@CM cell connected with the external resistors of 1000 Ω for cycling measurements.

**Table S1.** Structural parameters extracted from the Cu *K*-edge EXAFS fitting.

| Sample | Path | *N* | *R* (Å) | *σ^2^* (10^−3^ Å^2^) | Δ*E*_0_ (eV) | *R* factor |
| --- | --- | --- | --- | --- | --- | --- |
| Ru-Cu NTs@CM | Cu-Cu | 12 | 2.534 ± 0.0108 | 9.1 ± 1.457 | 3.57 ± 1.64 | 0.0269 |
| Cu NWs@CM | Cu-Cu | 12 | 2.538 ± 0.00589 | 8.74 ± 0.788 | 4.135 ± 0.896 | 0.00819 |

*N* is the coordination number; *R* is interatomic distance (the bond length between Cu central atoms and surrounding coordination atoms); *σ^2^* is Debye-Waller factor (represents the thermal and static disorder in absorber-scatterer distances); Δ*E*_0_ is edge-energy shift (the difference between the zero kinetic energy value of the sample and that of the theoretical model). *R* factor is used to assess the goodness of the fitting.

**Table S2.** Comparison of HER catalytic performance of Ru-Cu NTs@CM with the recently reported Ru or Cu based electrocatalysts in 0.5 M H_2_SO_4_.

| Electrocatalyst | *η*_10_  (mV) | Tafel slope  (mV dec^−1^) | Reference |
| --- | --- | --- | --- |
| *Ru-Cu NTs@CM* | *17* | *54.6* | *This work* |
| RuO_2_-WC NPs | 58 | 66 | *Angew. Chem. Int. Ed.* **2022**, *61*, 202202519 |
| Ru_SA_@NiFe PPc | 40 | - | *Energy Environ. Sci.* **2024**, *17*, 1540 |
| DNTs-Cu | 61 | 33 | *Nat. Mater.* **2025**, *24*, 424 |
| RuCo/TiO_2_ NTs | 17 | 18 | *Adv. Energy Mater.* **2024**, *15*, 2403067 |
| Ru/ZnRuO_2_ | 97 | 51.5 | *Adv. Funct. Mater.* **2024**, *34*, 2409306 |
| Ru_3_Sn_7_ | 28 | 22 | *Adv. Mater.* **2023**, *35*, 2302007 |
| Ru/NC-400 | 55 | 39 | *Adv. Funct. Mater.* **2021**, *31*, 2100698 |
| UP-RuNi_SAs_/C | 18 | 42.9 | *Nat. Commun.* **2024**, *15*, 2218 |
| Ru@WNO-C | 172 | 38.9 | [*Nano Energy*](https://www.sciencedirect.com/journal/nano-energy) **2021**, *80*, 105531 |
| Ru-OC_60_-300/KB | 53 | - | *ACS Catal.* **2023**, *13*, 7597 |
| Ru/TiO_2_/NC | 52 | 22.4 | *Small* **2024**, *20*, 2311667 |
| Ru-VO_2_ | 46 | 39.1 | *Adv. Mater.* **2024**, *36, 2310690* |
| Cu@Cu_3_P-Ru/CCG-500 | 102.52 | 66.4 | *‌Appl. Catal. B Environ.* **2023**, *326*, 122402 |
| Ru@V-RuO_2_/C HMS | 47 | 55.1 | *Adv. Mater.* **2023**, *35*, 2206351 |
| Ru-VO_2_ | 46 | 39.1 | *Adv. Mater.* **2024**, *36*, 2310690 |
| Ru/RuS_2_ | 45 | 24.4 | *Angew. Chem. Int. Ed.* **2021**, *60*, 12328 |

**Table S3.** Comparison of FOR catalytic performance of Ru-Cu NTs@CM with the recently reported electrocatalysts in alkaline media.

| Electrocatalyst | Electrolyte | *E*_100_ (mV vs. RHE) | Reference |
| --- | --- | --- | --- |
| *Ru-Cu NTs@CM* | *1 M KOH & 0.6 M HCHO* | *126* | *This work* |
| Cu_x_O@CF | 1 M KOH & 0.1 M HCHO | 136 | *Chem* **2023**, *9*, 963 |
| Pt_1_/Cu-CF | 1 M KOH & 0.5 M HCHO | ~ 50 | *Energy Environ. Sci.* **2025**, *18*, 6106 |
| Cu_3_Ag_7_/CF | 1 M KOH & 0.6 M HCHO | 100 | *Nat. Commun.* **2023**, *14*, 525 |
| CuFe | 1 M KOH & 0.2 M HCHO | 100 | *Adv. Funct. Mater.* **2024**, *35*, 2417545 |
| CuO/Cu_2_O@CF | 1 M KOH & 0.6 M HCHO | ~ 126 | *Angew. Chem. Int. Ed.* **2025**, *64*, 202504894 |
| CF@Cu-NS | 1 M KOH & 0.05 M HCHO | ~ 350 | *Angew. Chem. Int. Ed.* **2023**, *62*, 202302950 |
| Ag_1_@Cu_2_ONWs | 1 M KOH & 0.2 M HCHO | 40 | *Energy Environ. Sci.* **2025**, *18*, 2804 |
| Cu_2_O | 1 M KOH & 1 M HCHO | 320 | *Energy Environ. Sci.* **2023**, *16*, 2696 |
| PdCu NP@CF | 1 M KOH & 0.1 M HCHO | 280 | *Nat. Commun.* **2024**, *15, 9852* |

**Table S4.** Comparison of FPFC to the state-of-the-art self-powered H_2_ production systems.

| System | Catalyst | Electrolyte | *FE*  (%) | OCV  (V) | *P*_max_  (mW cm^−2^) | Reference |
| --- | --- | --- | --- | --- | --- | --- |
| *FOR-HER* | *Ru-Cu NTs@CM\|\|Ru-Cu NTs@CM* | *1 M KOH & 0.6 M HCHO\|\|0.5 M H_2_SO_4_* | *200* | *1.03* | *18.3* | *This work* |
| FOR-NO_3_RR | RhCu NW/CF\|\|RhCu NW/CF | 1 M KOH & 0.1 M HCHO\|\|1 M KOH & 0.1 M KNO_3_ | 100 | 0.56 | 10.76 | *Angew. Chem. Int. Ed.* **2025**, *64*, 202503424 |
| FOR-ORR | Cu/CF\|\|Pt/C | 2 M KOH & 0.4 M furfural\|\|humidified O_2_ | 100 | 0.96 | 193 | *Adv. Funct. Mater.* **2024**, *34*, 2404105 |
| HzOR-HER | RuTe_2_-Ru@CNFs\|\|RuTe_2_-Ru@CNFs | 1 M KOH & 0.5 M N_2_H_4_\|\|0.5 M H_2_SO_4_ | 100 | 0.95 | 17.1 | *Appl. Catal. B: Environ. Energy* **2024**, *358*, 124414 |
| Zn-HER | Zn\|\|Ce-Ni-BDC@NF@Pt/C-CP | 1 M KOH & 0.02 M Zn(CH_3_COO)_2_\|\|1 M KOH & 0.5 M urea | 100 | 1.22 | 2.32 | *Adv. Funct. Mater.* **2025**, 17562 |
| Mg-HER | Mg\|\|  Ni/V_2_O_3_@NF | 3.5% NaCl | 100 | 1.16 | 17.81 | *Chem. Eng. J.* **2022**, *450*, 138079 |
